# P301S‐hTau acetylates KEAP1 to trigger synaptic toxicity via inhibiting NRF2/ARE pathway: A novel mechanism underlying hTau‐induced synaptic toxicities

**DOI:** 10.1002/ctm2.1003

**Published:** 2022-08-02

**Authors:** Jia‐Zhao Xie, Yao Zhang, Shi‐Hong Li, Hui Wei, Hui‐Ling Yu, Qiu‐Zhi Zhou, Lin‐Yu Wei, Dan Ke, Qun Wang, Ying Yang, Jian‐Zhi Wang

**Affiliations:** ^1^ Department of Pathophysiology School of Basic Medicine, Key Laboratory of Education Ministry of China/Hubei Province for Neurological Disorders Tongji Medical College Huazhong University of Science and Technology Wuhan China; ^2^ Endocrine Department of Liyuan Hospital Key Laboratory of Education Ministry of China/Hubei Province for Neurological Disorders Tongji Medical College Huazhong University of Science and Technology Wuhan China; ^3^ Co‐Innovation Center of Neuroregeneration Nantong University Nantong China

**Keywords:** acetylation, KEAP1, NRF2, oxidative stress, P301S, synapse loss

## Abstract

**Background:**

Human Tau (hTau) accumulation and synapse loss are two pathological hallmarks of tauopathies. However, whether and how hTau exerts toxic effects on synapses remain elusive.

**Methods:**

Mutated hTau (P301S) was overexpressed in the N2a cell line, primary hippocampal neurons and hippocampal CA3. Western blotting and quantitative polymerase chain reaction were applied to examine the protein and mRNA levels of synaptic proteins. The protein interaction was tested by co‐immunoprecipitation and proximity ligation assays. Memory and emotion status were evaluated by a series of behavioural tests. The transcriptional activity of nuclear factor‐erythroid 2–related factor 2 (NRF2) was detected by dual luciferase reporter assay. Electrophoresis mobility shift assay and chromosome immunoprecipitation were conducted to examine the combination of NRF2 to specific anti‐oxidative response element (ARE) sequences. Neuronal morphology was analysed after Golgi staining.

**Results:**

Overexpressing P301S decreased the protein levels of post‐synaptic density protein 93 (PSD93), PSD95 and synapsin 1 (SYN1). Simultaneously, NRF2 was decreased, whereas Kelch‐like ECH‐associated protein 1 (KEAP1) was elevated. Further, we found that NRF2 could bind to the specific AREs of DLG2, DLG4 and SYN1 genes, which encode PSD93, PSD95 and SYN1, respectively, to promote their expression. Overexpressing NRF2 ameliorated P301S‐reduced synaptic proteins and synapse. By means of acetylation at K312, P301S increased the protein level of KEAP1 via inhibiting KEAP1 degradation from ubiquitin–proteasome pathway, thereby decreasing NRF2 and reducing synapse. Blocking the P301S–KEAP1 interaction at K312 rescued the P301S‐suppressed expression of synaptic proteins and memory deficits with anxiety efficiently.

**Conclusions:**

P301S‐hTau could acetylate KEAP1 to trigger synaptic toxicity via inhibiting the NRF2/ARE pathway. These findings provide a novel and potential target for the therapeutic intervention of tauopathies.

## Graphical abstract

P301S‐hTau induces synapse loss via dysregulation of KEAP1/NRF2 pathway because it induces an increase of KEAP1 and a decrease of NRF2, and NRF2 transcriptionally regulates the expression of synaptic proteins.

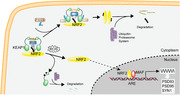

## 1 INTRODUCTION

Tauopathies constitute a subset of neurodegenerative diseases, such as Alzheimer's disease (AD) and frontotemporal dementia with parkinsonism linked to chromosome 17 frontotemporal dementia (FTDP‐17), which are characterized by the aggregation of human Tau (hTau) protein within the neurons.^1^, ^2^, ^3^ The severity of neurodegeneration and cognitive deficits has a close correlation with the deposition of abnormal hTau aggregates in the brain.^4^ Importantly, Braak staging, which analyses neurodegeneration‐associated neuropathology and generates an algorithm of staging to describe Tau anatomical distribution, has been identified to be a useful method for examining the alternations of molecular and pathological events in the brains of AD patients.^5^, ^6^, ^7^

Accumulating studies have revealed an initial role of hTau pathologies in neurodegenerative disease.^8^, ^9^ In P301S mutation cases, the mean age of clinical onset and death was reported as early as ∼34.3 and ∼40.8‐year old, respectively.^10^ As clinical symptoms developed, the temporal atrophy rate was detectably increased in P301S‐carried patients, followed by atrophy of the parietal and frontal lobes.^11^ Compared with AD patients, brain atrophy progresses more rapidly in P301S‐carried patients,^12^ suggesting that mutated hTau is more toxic than wild‐type hTau in neurodegeneration. In PS19 mice carrying mutated P301S, the neurological deficits of tauopathies with dementia and increased pre‐pulse inhibition (PPI), which is a characteristic of amnestic mild cognitive deficit, were well recapitulated.^13^ Importantly, synaptic dysfunction rather than neuron loss and neurofibrillary tangle formation was detectable at as early as 3 months of age in PS19 mice,^14^ indicating an initial role of hTau in synaptic dysfunction during the process of neurodegeneration. However, the underlying mechanism is still unclear.

Oxidative stress, a result of imbalance in reactive oxygen species (ROS) production and antioxidative capacity,^15^ is another early event during the development of neurodegenerative disease.^16^ Kelch‐like ECH‐associated protein 1 (KEAP1)‐nuclear factor‐erythroid 2–related factor 2 (NRF2) is an important intracellular antioxidant pathway that maintains intracellular homeostasis.^17^ NRF2 is a major regulator of anti‐oxidative response element (ARE)‐driven cytoprotective gene. However, KEAP1 is a suppressor of NRF2 and acts as an adaptor protein for a Cul3‐based E3 ligase.^18^, ^19^ Upon oxidative or electrophilic stress, cytoplasmic NRF2 translocates into the nucleus after dissociating from KEAP1 and binds to ARE regions of targeted genes to activate gene transcription.^20^ A transcriptomic analysis demonstrated that seven and ten of the most dysregulated functional pathways during ageing and AD were both replicated within the brain of NRF2‐knockout mice.^21^ Moreover, both mRNA and protein levels of NRF2 are reduced, whereas the mRNA of KEAP1 is increased in some neurodegenerative diseases such as motor cortex of amyotrophic lateral sclerosis.^22^ It has been reported that NRF2 in astrocytes exerts protective effects against optic tract damage during the process of cerebral hypoperfusion.^23^ Other reports suggest that NRF2 activity in astrocytes and microglia is important for the prevention of AD progression.^24^ However, whether and how hTau modifies the KEAP1/NRF2 pathway and contributes to hTau‐induced synapse loss are elusive.

In this study, we observed that overexpressing P301S significantly decreased the expression of synaptic proteins with an impaired KEAP1/NRF2 pathway. NRF2 could promote the expression of synaptic proteins via binding to the different specific ARE element of the DLG2, DLG4 and SYN1 genes. Upregulating NRF2 attenuated P301S‐induced synapse loss and cognitive dysfunction, whereas downregulating NRF2 replicated synaptic pathology and malfunction. We also found that P301S interacted with KEAP1 and acted as an acetyltransferase to promote the acetylation of KEAP1 at K312, by which it inhibited the ubiquitination of KEAP1 and slowed its degradation. Blocking the interaction between P301S and KEAP1 at K312 by a custom‐designed peptide could efficiently rescue P301S‐induced synaptic protein reduction and cognitive deficits in a manner dependent on NRF2 expression.

## 2 MATERIALS AND METHODS

### 2.1 Animals

Two‐month‐old male C57BL/6J mice and pregnant Sprague–Dawley (SD) rats were purchased from the animal centre of Tongji Medical College, Huazhong University of Science and Technology. Animals were housed at 22 ± 2°C with ad libitum access to food and water on a 12 h light–dark cycle. All animal experiments were performed according to the ‘Policies on the Use of Animals and Humans in Neuroscience Research’ revised and approved by the Society for Neuroscience in 1995, and the Guidelines for the Care and Use of Laboratory Animals of the Ministry of Science and Technology of the People's Republic of China, and the Institutional Animal Care and Use Committee at Tongji Medical College, Huazhong University of Science and Technology approved the study protocol.

### 2.2 Reagents

BCA assay (BL521A), enhanced chemiluminescent (ECL) substrates (BL523A) nuclear extract kit (BL670A) and DAPI (BL739B) were purchased from Biosharp (Beijing, China). B27 (17504044), trypsin (15050057), poly‐d‐lysine (A3890401), 1% penicillin–streptomycin (15140163), fetal bovine serum (FBS) (10099141), neurobasal medium (21103049), DMEM/F12 (11320033), MEM (12492013), TRIzol reagent (15596018) and trypsin (25200072) were purchased from Thermo Fisher (MA, USA). SiRNA for KEAP1 was purchased from AUGCT (Beijing, China). EGFP‐N1 vector‐coded wild‐type hTau (2N4R), mutated P301S (1N4R), mutated P301L (2N4R), pmCherry‐C1 vector‐coded NRF2, p3xFLAG vector‐coded KEAP1 and pX459 vector‐coded single‐guild RNA against mouse NRF2 plasmids (sgNRF2) were prepared in our lab. The nonradioactive NRF2 electrophoresis mobility shift assay (EMSA, GS‐0031) kit and pNRF2‐Luc (LR‐2106) were purchased from Signosis (CA, USA). Dihydroethidium (DHE) fluorescence probe (S0630), Dual‐Lumi dual luciferase reporter (DLR) gene assay kit (RG088S) and chromosome immunoprecipitation (ChIP) kit (P2078) were purchased from Beyotime (Shanghai, China). MG132 (HY‐13259) and cycloheximide (CHX) (HY‐12320) were products of MedChemExpress (NJ, USA). Peptide P1: CDVTLQVKYQDAPAA (block K84 of KEAP1), P2: FEELTLHKPTQAVPC (block K312 of KEAP1) were synthesized by Bioyeargene (Wuhan, China). Neofect DNA transfection reagent (TF201201) was purchased from Neofect (Beijing, China). Protein A+G agarose (36403ES03), SYBR mix (11203ES03), Hifair^@^ II 1st Strand cDNA Synthesis Kit (11121ES60) were products of Yeasen (Shanghai, China). An FD rapid Golgi Stain kit was purchased from FD NeuroTechnologies (USA). Antibodies and its application were listed in Table S1.

### 2.3 Stereotaxic brain injection of AAV virus

Adeno‐associated virus encoding mutated hTau P301S (AAV2/9‐P301S‐EGFP), human full length NRF2 (AAV2/9‐NRF2‐mCherry), AAV2/9‐Cas9 and AAV2/9‐sgNRF2‐Cas9 were purchased from OBiO Technology (Shanghai, China). AAV2/9‐P301S‐EGFP and AAV2/9‐NRF2‐mCherry titres were 5×10^12^ vg/ml, and those of AAV2/9‐Cas9 and AAV2/9‐sgNRF2‐Cas9 viruses were 1×10^13^ vg/ml. For hippocampal injection, 2‐month‐old C57BL/6 mice were placed in a stereotaxic instrument (RWD68001, Shenzhen, China) after anaesthesia, and the virus was injected bilaterally into the CA3 region of hippocampal (AP ±2.5, DV −2.0, ML –2.0) at a rate of 1 μl/10 min. After injection, hold for 10 min and withdraw the syringe needle.

### 2.4 In vivo peptide administration

Four weeks after AAV2/9‐P301S‐EGFP injection in the hippocampal CA3 region, C57BL/6 mice were repeatedly administered 5‐mM custom‐designed peptide (Bioyeargene, Wuhan, China) via lateral ventricle‐implanted guiding cannulas (RWD, Shenzhen, China) once every 3 days for 4 weeks to test its blocking efficiency. During dosing, mice were restricted by a stereotaxic apparatus and kept awake. All mice were sacrificed 24 h after the last administration.

### 2.5 Cell culture of neuroblastoma 2a and primary neuron

Mouse neuroblastoma 2a (N2a, supplied by the China Center for Type Culture Collection) was grown in MEM (10% FBS and 1% penicillin/streptomycin) in a humidified incubator (HF90, Healforce, China) at the condition of 5% CO_2_ at 37°C. When grown to 70% confluency in plates, the N2a cells were transfected with plasmids using a Neofect DNA transfection reagent.

Isolation and culture of primary hippocampal neurons was prepared according to the published literature.^25^ Briefly, pregnant SD rats with E15–E18 days embryos were euthanized, and embryos were removed from the blastocysts of pregnant rats and then the hippocampus was isolated on ice, minced in DMEM/F12 and digested in .125% (vol/vol) trypsin solution for 20 min at 37°C. Digestion was terminated by adding FBS. Neurons were cultured in FBS‐containing DMEM/F12 (10%) for 6 h, and subsequently cultured in neurobasal medium (2% B27 and 1% penicillin–streptomycin) in pre‐coated culture plates. Every 3 day, the half volume of medium was replaced by fresh medium. The purity of neurons was >97% under this condition in our lab. The number of neurons in the 6‐well culture plate was approximately 1 × 10^5^ cells/well for Western blotting or quantitative polymerase chain reaction (qPCR) analysis, and approximately 1 × 10^3^ cells/well for IF.

### 2.6 Behavioural tests

After virus injection or peptide administration, cognitive capacity was evaluated by the novel object recognition (NOR), the Morris water maze (MWM) and the fear conditioning test (FCT), respectively. However, the emotional status was detected by the open field test (OFT) and elevated plus maze (EPM).^26^

### 2.7 Nuclear fractionation of cells or tissue preparation

The nuclear extracts of cells or tissues were prepared according to the instruction of the nuclear extract kit. Briefly, cells were washed with pre‐cool phosphate buffer saline (PBS) and collected by centrifugation for 5 min at 800 × *g*. Cell pellets were reserved and resuspended in solution I (450‐μl A, 50‐μl B, 1‐mM Phenylmethanesulfonyl fluoride (PMSF). When extracting nuclear extract, tissues were cut into 1‐mm^3^ pieces, added solution I working reagent and homogenized on ice. For cells, a solution I working reagent was added into cell plate after washing with PBS then incubated on ice for 30 min and collected into a tube. The supernatant was removed after centrifugation at 10000 × *g* for 10 min at 4°C, and the cells or tissue sediment were resuspended with a Buffer C working reagent, and rocked at 200 rpm on a shaking platform for 10 min at 4°C. The supernatant was collected after centrifugation and analysed by Electrophorisis mobility shift assay (EMSA) or Western blotting.

### 2.8 Electrophoresis mobility shift assay

The nuclear extracts from cells were incubated with a biotin‐labelled probe (containing an ARE motif for NRF2 binding) for 30 min at room temperature. The mixtures were loaded and separated by a non‐denaturing polyacrylamide gel. After being transferred to a nylon membrane, the probe–NRF2 complex was fixed by ultraviolet cross‐linking for 10 min at 120 000 J. Then the membrane was incubated with streptavidin‐HRP (1:1000) for 1 h at RT, and the blots on the membrane were visualized by ECL solution in a chemiluminescence apparatus (ChemiScope 6000, Clinx, China). ImageJ (NIH, USA) was used to analyse bands on the membrane. Competition experiment was done by adding excess unlabelled cold probe.

### 2.9 Dual luciferase reporter assay

Briefly, the P301S plasmid or vector, pNRF2‐Luc reporter construct and Renilla luciferase construct (pRL‐TK) were transfected into HEK293T cells using a Neofect DNA transfection reagent. After 24 h, cells were harvested and washed with precool PBS three times and lysed with firefly luciferase detection buffer (100 μl). The transfection efficiency of pRL‐TK was applied to normalize the activity of NRF2. Luciferase activity assay of cell extracts was performed in the DLR Assay System (Promega, WI, USA).

ARE fragments of the DLG2, DLG2 and SYN1 promoter were cloned into the pGL3 luciferase reporter vector (Promega, WI, USA). The mutation site of the pGL3‐DLG2/DLG4/SYN1 luciferase plasmids was constructed by the GeneTailor system (Invitrogen, USA). pGL3‐DLG2/DLG4/SYN1 luciferase plasmids were transfected into HEK293T cells using a Neofect DNA transfection reagent. An adequate quantity of HEK293T cells was cultured into cultured plates before transfection and then transfected with P301S or vector, pGL3 constructs and pRL‐TK plasmids. HEK293T cells were collected 24 h later and lysed with a firefly luciferase detection buffer (100 μl/well). The luciferase activity of each plasmid was determined using 20‐μl cell extracts in the DLR Assay System (Promega, WI, USA).

### 2.10 Golgi‐cox staining and synaptic spine analysis

The Golgi‐cox staining procedure was performed according to the protocol. Briefly, the brains of mice were removed after anesthetization using 2% pentobarbital sodium and immersed in a mixture of Solutions A + B for 2 weeks at RT in dark. Then the brains were transferred into a new tube and added Solution C for another 7 days at 4°C in dark. All brains were sliced into 100‐μm‐thick sections using a Vibratome (VT1200S; Leica, Germany). For Sholl analysis, brain slice images were obtained with a microscope (VS120, Olympus, Japan), and dendritic spines were imaged with an oil microscope (Ni‐E, Nikon, Japan). Sholl analysis and spine number were analysed using ImageJ (NIH, USA).

### 2.11 Chromatin immunoprecipitation (ChIP) assay

Cell samples for the ChIP assay were treated according to the previously described methods.^27^ The DNA and protein cross‐link was finished using formaldehyde (1%) for 15 min, and the mixture reaction was abolished by glycine solution, then washed with PBS, and scraped with cold PBS (1‐mM PMSF). Collected and resuspended the cell pellet with SDS lysis buffer (1‐mM PMSF) after centrifugation and then incubated on ice for 20 min. Sonicated cell pellet for 5–10 s and centrifuged at 14 000 × *g* for 10 min at 4°C. Transferred the supernatant into a new tube and added 2‐ml ChIP dilution buffer. A volume of 20‐μl supernatant was reversed as the input, and NRF2 antibody or IgG was added to the remainder for IP, and incubated with protein A + G agarose, and rotated for 12 h at 4°C. Centrifugated the mixtures at 1000 × *g* for 6 min then rinsed pellet once with low salt, high salt‐immune complex wash buffer and LiCl immune complex wash buffer once, respectively. TE buffer was used to rinse the pellet twice at last. Vortexed the pellet after adding a 250‐μl elution buffer. To remove cross‐linking between DNA and protein, samples were added with 5‐M NaCl and incubated in a water bath at 65°C for 4 h. Then 10‐μl EDTA (.5 M), 20‐μl Tris (1 M, pH 6.5) and 1‐μl proteinase K (20 mg/ml) were added and vortexed and incubated for 1 h at 45°C in a water bath. After isolated using phenol/chloroform extraction, the pellet was added 20‐μg glycogen, 1/10 (v/v) 3‐M NaAc (pH 5.2) and 2.5 times the volume of absolute ethanol. After vortexing, the mixtures were placed at −80°C for at least 8 h. The mixture was centrifuged at 4°C, and 14 000 × *g* for 10 min. The DNA in the pellet was resuspended with TE buffer. The ChIP PCR primers are listed in Table S3. The products were analysed by quantitative PCR. The specific ARE fragments of the DLG2, DLG4 and SYN1 for NRF2 binding were predicted by JASPAR (https://jaspar.genereg.net/), and NFE2LE (gene name of NRF2) was chosen as a transcriptional regulator. Then, the promoter sequence of the DLG2/DLG4/SYN1 (from −2000 to 200 bp of start codon) in a FASTA format was put into the scan block in a FASTA format. The relative profile score threshold was set to approximately 80% or lower according to the scan results. Predicted ARE sequences are listed according to the calculated score, and a higher score means a higher interaction. Three to four predicted sites were chosen as candidates for validation by qPCR. For DLG2, three predicted sequences were chosen as candidates (−1417 to −1410, −336 to −328, −125 to −117). For DLG4, one predicated sequence was chosen as a candidate (−1054 to −1046). For SYN1, three predicated sequences were chosen as candidates (−974 to −966, −634 to −626, −137 to −130).

### 2.12 Statistical analysis

All data are expressed as the mean ± SEM and were analysed in a blinded manner by other researchers. All data were analysed and plotted using Prism 8.0 (GraphPad Software). Statistical analysis was performed using unpaired Student's *t*‐test in two‐group comparisons, one‐way analysis of variance (ANOVA) by Bonferroni's post hoc test or two‐way ANOVA followed by Bonferroni's post hoc test as illustrated in each figure legend. The normality of the data distribution was confirmed by the D'Agostino–Pearson omnibus normality test and using the *F*‐test, Brown–Forsythe test and Bartlett's test. All the data fit homogeneity of variance. A *p* value <.05 was considered as statistically significant in all experiments.

## 3 RESULTS

### 3.1 Overexpressing P301S promotes oxidative stress and downregulates NRF2 proteins

**FIGURE 1:**
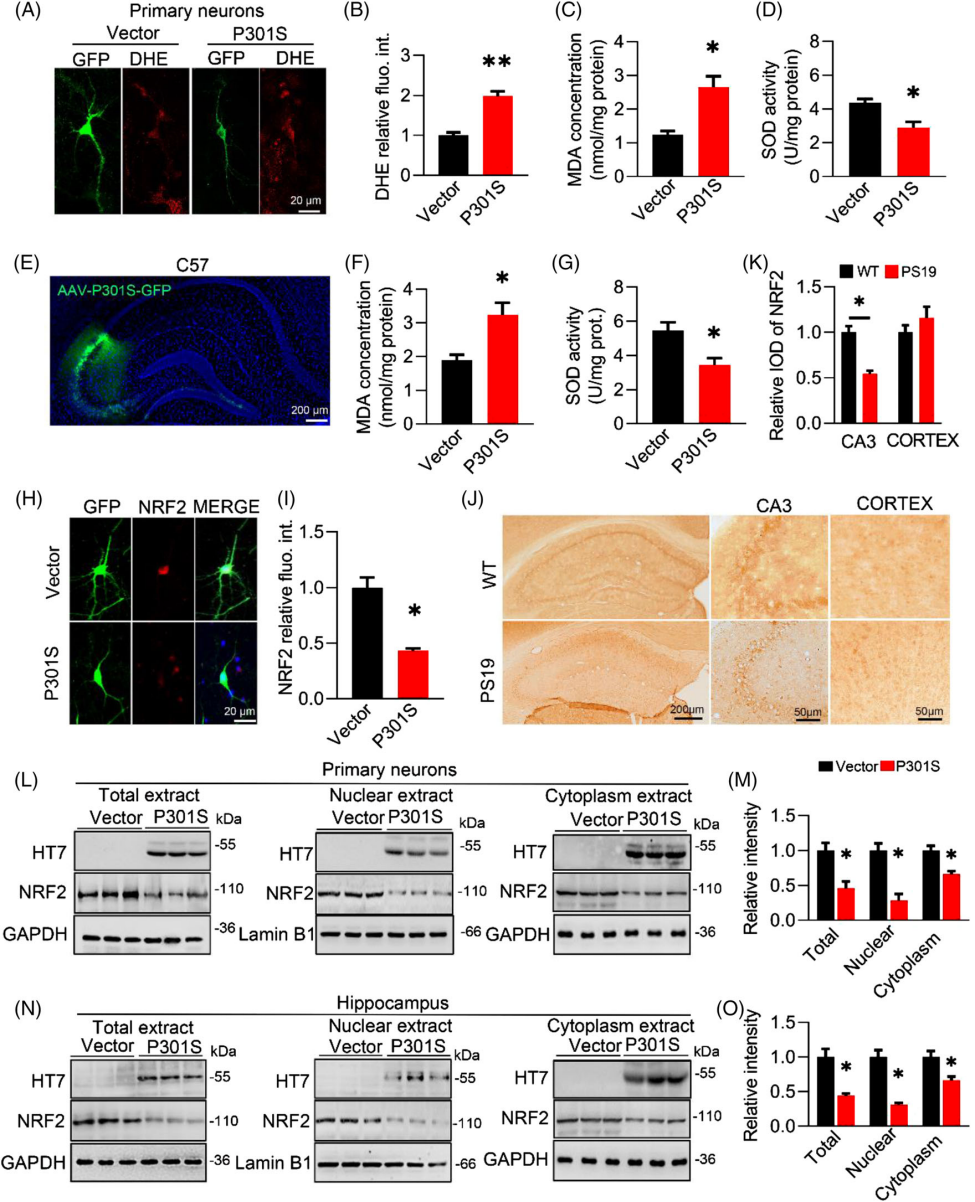
P301S accumulation promotes oxidative stress and downregulates nuclear factor‐erythroid 2–related factor 2 (NRF2) with an inhibited nuclear translocation. (A and B) Overexpressing P301S (GFP‐label) in primary hippocampal neuron increased reactive oxygen species (ROS) level as indicated by dihydroethidium (DHE) probe (red). Unpaired *t*‐test, *t* = 5.463 df = 4, *p* = −.0043; *n* = 3. (C and D) Overexpressing P301S reduced malondialdehyde (MDA) concentration (C) while decreased superoxide dismutase (SOD) activity (D) in primary hippocampal neuron. Unpaired *t*‐test, [MDA] *t* = 4.016 df = 4, *p* = .0159; [SOD] *t* = 3.515 df = 4, *p* = .0246; *n* = 3. (E–G) Overexpressing P301S reduced MDA concentration (C) while decreased SOD activity (D) in CA3. Unpaired *t*‐test, [MDA] *t* = 4.874 df = 4, *p* = .0183; [SOD] *t* = 5.342 df = 4, *p* = .0139; *n* = 3. (H and I) Overexpressing P301S reduced NRF2 protein level examined by immunofluorescence. Unpaired *t*‐test, *t* = 4.434 df = 4, *p* = .0239; *n* = 3. (J and K) Decreased NRF2 was detected in the CA3 of 8‐month‐old PS19 mice compared with wild‐type (WT) mice. Unpaired *t*‐test, *t* = 5.542 df = 4, *p* = .0351; *n* = 3. (L and M) Overexpressing P301S decreased nuclear factor‐erythroid 2–related factor 2 (NRF2) protein level in total, nuclear and cytoplasm extracts of primary neurons. Unpaired *t*‐test, [total NRF2] *t* = 3.655 df = 4, *p* = .0215; [nuclear NRF2] *t* = 4.063 df = 4, *p* = .0153; [cytoplasm NRF2] *t* = 4.982 df = 4, *p* = .0415; *n* = 3. (N and O) Overexpressing P301S decreased NRF2 protein level in total, nuclear and cytoplasm extracts of C57BL/6J hippocampus. Unpaired *t*‐test, [total NRF2] *t* = 3.733 df = 4, *p* = .0202; [nuclear NRF2] *t* = 4.103 df = 4, *p* = .0148; [cytoplasm NRF2] *t* = 4.571 df = 4, *p* = .0412; *n* = 3. VEC, vector control. * *p* < .05, ** *p* < .01. Data were presented as mean ± SEM.

To investigate the causal role of P301S in oxidative stress, we first transfected N2a cells with plasmid to overexpress P301S exogenously. After 48 h, we found that ROS detected by the DHE probe was significantly increased in the P301S group as compared with the vector control (Figure S1a,b). Simultaneously, the concentration of malondialdehyde (oxide, MDA) was increased (Figure S1c), whereas the activity of total superoxide dismutase (SOD, including SOD1/2/3) was decreased (Figure S1d). Then, we introduced exogenous P301S into primary hippocampal neurons (Figure 1A) and the hippocampus of C57BL/6J mice (Figure 1E) to further explore the toxic effect of P301S accumulation in vitro and in vivo. Again, DHE staining (Figure 1B) and MDA (Figure 1C,F) were significantly increased, whereas SOD (Figure 1D,G) was robustly decreased in the P301S group compared with the vector group. These data demonstrated that P301S accumulation induces oxidative stress with mechanisms involving an imbalanced pro‐ and anti‐oxidation response.

NRF2, a member of the NF‐E2‐related factor family, is a transcription factor that regulates the expression of antioxidant genes. By immunofluorescence, we found an obvious reduction of the intracellular NRF2 proteins in primary hippocampal neurons with P301S overexpression (Figure 1H,I). In addition to the whole extracts from primary neurons, the protein level of NRF2 in the nuclear and cytoplasm fractions was dramatically decreased, as shown by Western blotting (Figure 1L,M). Interestingly, NRF2 in the hippocampus, not the cortex, was significantly decreased in PS19 mice compared with WT mice by immunostaining (Figure 1J,K), suggesting the vulnerability of hippocampal NRF2 in response to P301S accumulation. To further confirm the inhibitory effect of P301S on NRF2 in vivo, we injected AAV2/9‐P301S into the hippocampal CA3 (one of the hippocampal subregions associated with emotion^28^ and spatial memory^29^) and performed Western blotting to examine the protein level of NRF2. Again, the reduction in NRF2 was obviously detectable in the whole extract, cytoplasm and nucleus (Figure 1N,O). Moreover, this reduction was NRF2 specific, as no significant alterations were found in NRF1 and NRF3 after P301S overexpression (Figure S1e,f). Immunofluorescence imaging in N2a cells also confirmed that overexpressing P301S decreased NRF2 proteins, especially its nuclear localization, compared to the vector (Figure S1g,h).

Given the reduction in NRF2 proteins, especially in the nuclear fraction, we next employed an EMSA and found that the combination of NRF2 with a specific ARE sequence in the P301S group was much less than that in the control group (Figure S1i,j). This combination could be blocked by a cold probe (Figure S1i,j). Furthermore, a DLR assay was conducted to examine the transcriptional activity of NRF2. A significant decrease in luciferase activity was detected in the P301S group compared with the vector group (Figure S1k). Additionally, the mRNA levels of NQO1 and GCLC, two target genes of NRF2, were significantly decreased in the presence of P301S (Figure S1l,m).

Together, these data demonstrate that along with oxidative stress, P301S reduces NRF2 proteins and inhibits its transcriptional activity.

### 3.2 Knocking down NRF2 phenotypes P301S‐induced oxidative stress and disorders of memory and emotion

**FIGURE 2:**
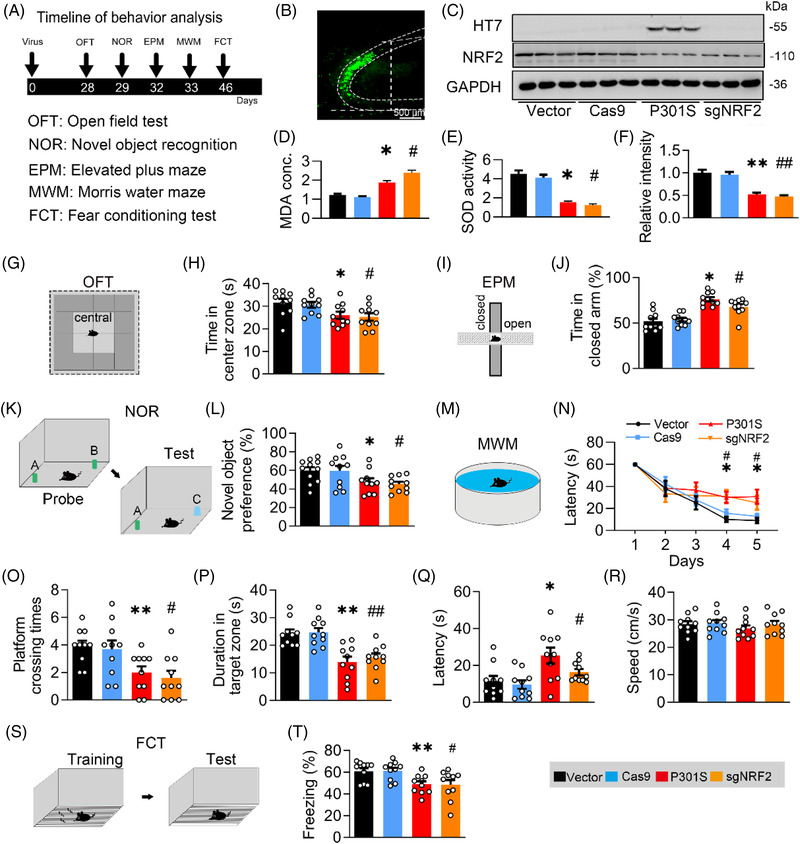
Knockdown nuclear factor‐erythroid 2–related factor 2 (NRF2) induces learning and memory deficits as seen in P301S mice. (A) Experimental procedures of virus injection and behavioural tests. AAV‐P301S‐EGFP or AAV‐VEC, AAV‐sgNRF2‐Cas9 or AAV‐Cas9 were stereotaxically injected into the hippocampal CA3 region of 2‐month‐old C57BL/6J mice. The capacity of learning and memory was detected 4 weeks later. (B) Efficiency of virus infection at CA3 was confirmed by GFP fluorescence. Bar, 500 μm. (C and F) The expression of P301s or NRF2 was confirmed by Western blotting. One‐way analysis of variance (ANOVA), *F*(3, 8) = 16.41, *p* =.0032; *n* = 3. (D and E) Overexpressing P301S increased malondialdehyde (MDA) concentration while reduced superoxide dismutase (SOD) activity in CA3 of C57BL/6J mice. One‐way ANOVA, [MDA] *F*(3, 8) = 23.13, *p* =.0081; [SOD] *F*(3, 8) = 19.53, *p* =.0043; *n* = 3. (G and H) Knockdown NRF2 decreased mice's staying time in centre zone of the box of open field test (OFT). One‐way ANOVA, *F*(3, 36) = 4.127, *p* = .0130; *n* = 10. (I and J) Knockdown NRF2 increased mice's staying time in frame of elevated plus maze (EPM) test. One‐way ANOVA, *F*(3, 36) = 19.23, *p* < .0001; *n* = 10. (K and L) Knockdown NRF2 impaired novel object recognition (NOR) as seen by overexpressing P301S. Cartoons show paradigms of NOR test. One‐way ANOVA, *F*(3, 37) = 3.479, *p* = .0254; *n* = 9–11 mice in each group. (M and N) Knockdown NRF2 induced spatial learning deficit as seen in P301S overexpressing measured by Morris water maze (MWM) test. Two‐way ANOVA × days, *F*(3, 180) = 4.116, *p* = .0075; *n* = 9–10 mice in each group. Cartoons show paradigms of MWM test. (O‐R) Knockdown NRF2, as seen by overexpressing P301S, impaired spatial memory shown by the decreased platform crossing times (O), decreased duration in the target quadrant (P) and an increased latency to reach the platform location (Q) in water maze detected at day 7 after removing the platform; the swimming speed was not changed by knockdown NRF2 or overexpressing P301S (R). One‐way ANOVA, [platform crossing times] *F*(3, 36) = 5.268, *p* = .0041; [duration] *F*(3, 36) = 11.76, *p* < .001. [Latency] *F*(3, 36) = 5.055, *p* = .005; *n* = 10 mice in each group. (S and T) Knockdown NRF2 impaired contexture memory, as seen in P301S overexpression, measured by fear conditioning test (FCT). One‐way ANOVA, *F*(3, 36) = 4.967, *p* = .0055; *n* = 10 mice in each group. * *p* < .05, ** *p* < .01 versus VEC; # *p* < .05, ## *p* < .01 versus Cas9. Data were presented as mean ± SEM.

To investigate whether and how NRF2 suppression affects cognitive capacity and emotion, we applied CRISPR‐Cas9 and designed a single‐guide RNA sequence (sgNRF2) into the hippocampal CA3 of 2‐month‐old C57BL/6J mice to knock down NRF2^30^ and performed a series of behavioural tests (Figure 2A,B). By Western blotting, the in vivo knockdown efficiency was 50%, which was similar to the level observed in P301S^CA3^ mice (exogenously overexpressing P301S hTau in the hippocampal CA3 subregion) (Figure 2C,F). Simultaneously, we detected a robust increase in MDA and a decrease in SOD in the sgNRF2 group (Figure 2D,E) suggesting oxidative stress induced by NRF2 reduction. Behaviourally, both sgNRF2 and P301S^CA3^ mice displayed anxiety, evidenced by less exploration time in the central zone of the OFT (Figures 2G,H and S2) and more investigation time in the closed arm of the EPM (Figures 2I,J and S3). Knocking down NRF2 significantly decreased novel object preference in the NOR test as observed in P301S^CA3^ mice (Figures 2K,L and S4). In the MWM test, both sgNRF2 and P301S^CA3^ groups showed relatively longer escape latency than controls during the 5‐day training set (Figure 2M,N). During the spatial memory test, the platform crossing times (Figures 2O and S5) and duration time in the target quadrant (Figure 2P) both decreased, whereas the escape latency (Figure 2Q) increased in the sgNRF2 and P301S^CA3^ groups, without a significant difference in swimming speed among the groups (Figure 2R). Knocking down NRF2 also significantly decreased freezing time in contextual FCT (Figure 2S,T). These data together indicate that knocking down NRF2 in the hippocampal CA3 mimics the toxic effect of P301S on memory and emotion.

**FIGURE 3:**
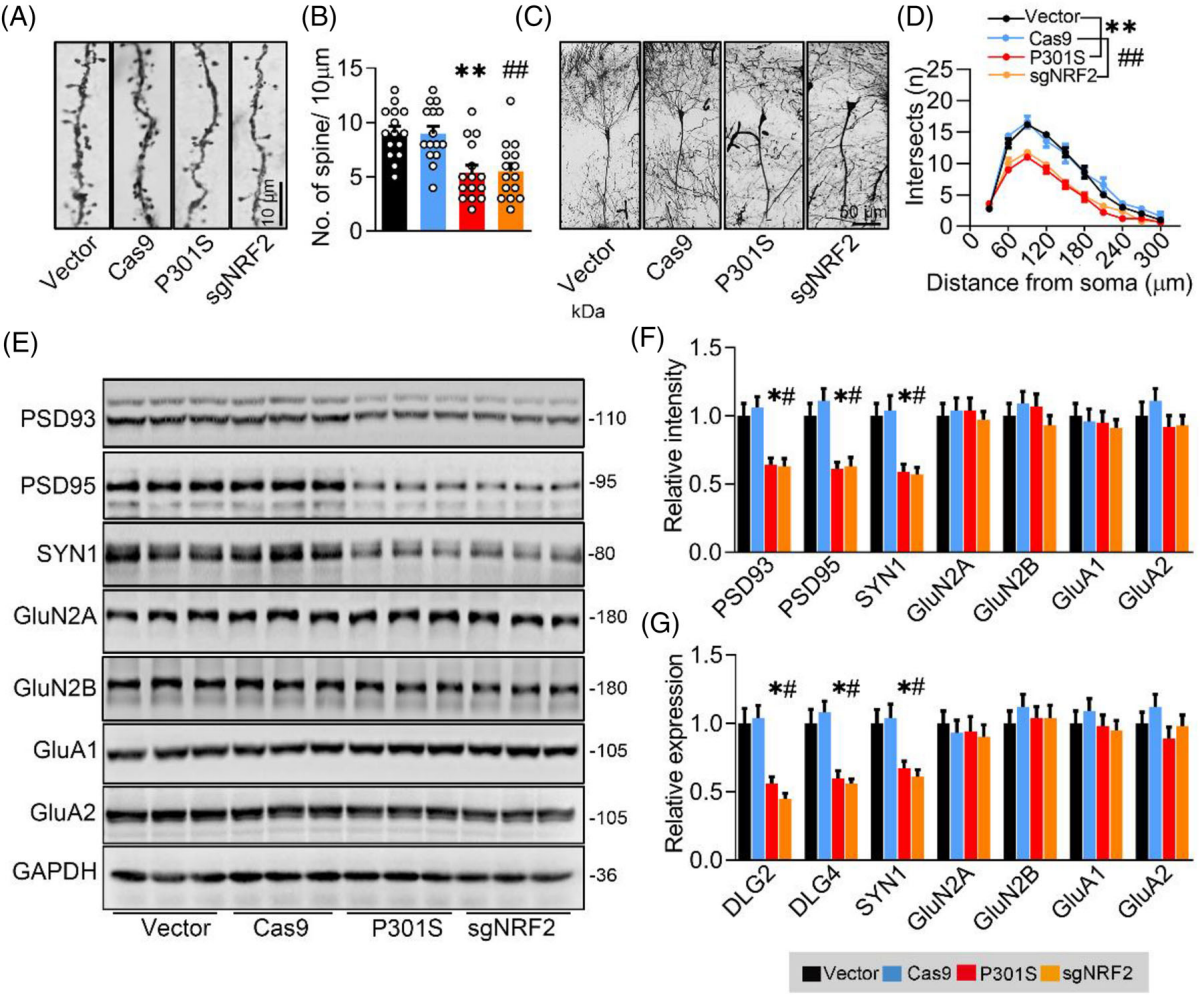
Knockdown nuclear factor‐erythroid 2–related factor 2 (NRF2) induces synapse loss and synaptic protein reduction as seen in P301S mice. (A and B) Knockdown NRF2 decreased spine densities as seen in P301S overexpression detected by Golgi‐cox staining. The representative images (A) and quantitative analysis (B) of spine. One‐way ANOVA), *F*(3, 56) = 9.896, *p* = .001, *n* = 15. (C and D) Sholl analysis showed synaptic complexity of neurons after knockdown NRF2 or overexpressing P301S. The representative images (C) and the quantitative analysis (D). Two‐way ANOVA × distance, *F*(3, 160) = 80.28, *p* = .0001; *n* = 5. (E–G) Knockdown NRF2 decreased protein (E and F) and mRNA levels (G) of synaptic proteins as seen in P301S overexpression. One‐way ANOVA, for panel (F) [post‐synaptic density protein 93 (PSD93)] *F*(3, 8) = 7.055, *p* = .0123; [PSD95] *F*(3, 8) = 5.867, *p* = .0203; [SYN1] *F*(3, 8) = 7.200, *p* = .0116; for panel (G) [DLG2] *F*(3, 8) = 15.09, *p* = .0122; [DLG4] *F*(3, 8) = 13.43, *p* = .0171; [SYN1] *F*(3, 8) = 7.296, *p* = .0112; *n* = 3. * *p* < .05, ** *p* < .01 versus VEC; # *p* < .05, ## *p* < .01 versus Cas9. Data were presented as mean ± SEM.

Next, we measured the synapse density and the expression of synaptic proteins among the groups. By Golgi‐cox staining, we detected lower spine density (Figure 3A,B) and less dendritic complexity in the sgNRF2 and P301S^CA3^ groups (Figure 3C,D), indicating synapse loss after NRF2 reduction. By Western blotting, we found a significant reduction in post‐synaptic density protein 93 (PSD93), PSD95 and SYN1 in the sgNRF2 and P301S^CA3^ groups compared with the control group (Figure 3E,F). No significant changes in GluN2A, GluN2B, GluA1 or GluA2 were observed among the groups (Figure 3E,F). These findings were replicated in the N2a cell line (Figure S6). Moreover, the mRNA levels of DLG2, DLG4 and SYN1 were dramatically and similarly reduced in the sgNRF2 and P301S^CA3^ groups (Figures 3G, and S6c,h). Interestingly, the synaptic toxicity of P301L and wild‐type hTau was quite different from that of P301S. Neither P301L nor wild‐type hTau decreased NRF2 protein level (Figure S7a,b). However, both significantly reduced the protein and mRNA levels of GluN2A and GluN2B (Figure S7a,b). These data not only reveal the difference in distinct hTau on synaptic proteins but also indicate a close association between NRF2 reduction and P301S‐induced synaptic toxicity.

Together, our data suggest that knocking down NRF2 could phenotype P301S‐induced oxidative stress and disorders of memory and emotion.

### 3.3 Upregulating NRF2 attenuates P301S‐induced synapse loss and disorders of memory and anxious status

**FIGURE 4:**
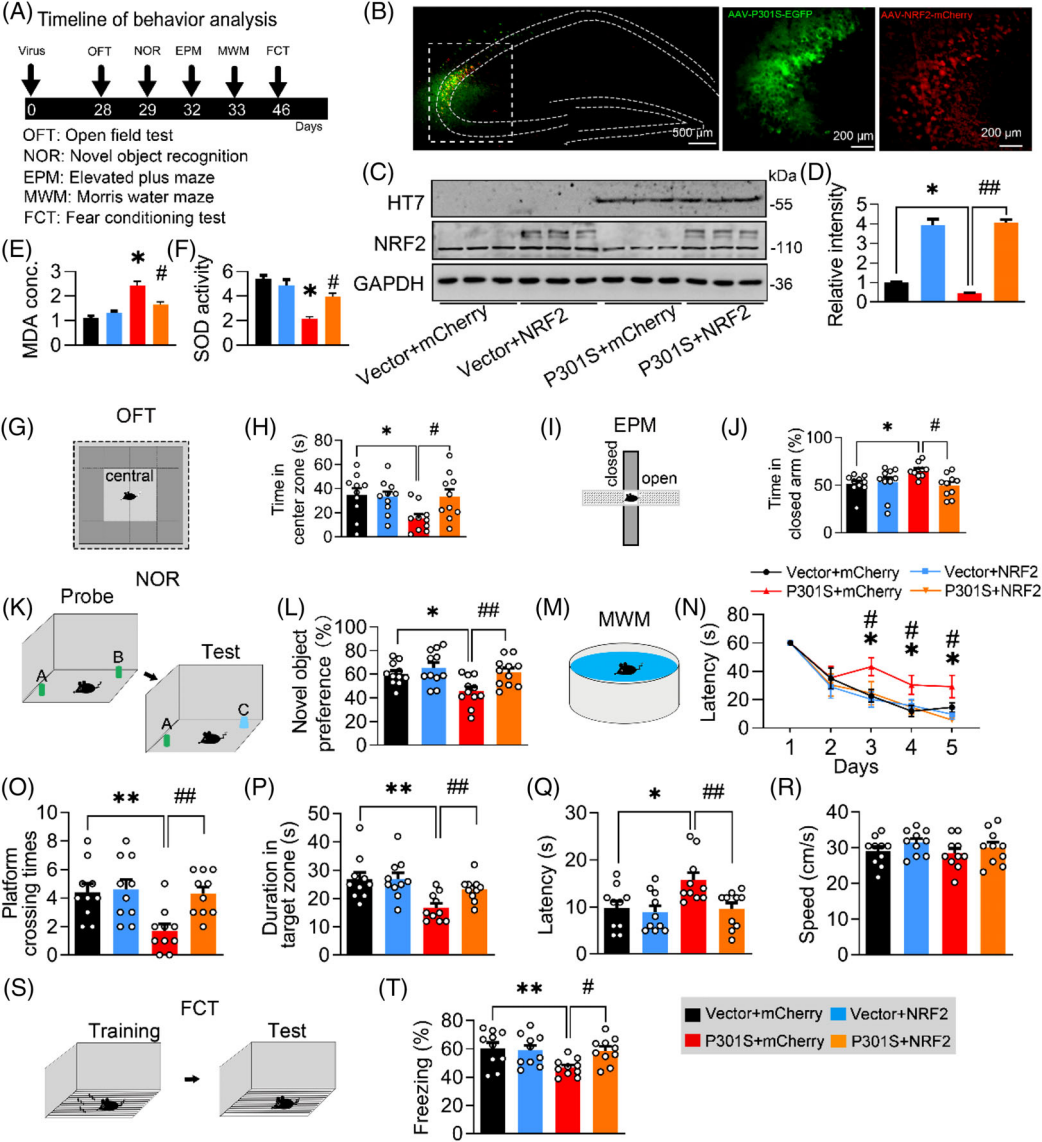
Overexpressing nuclear factor‐erythroid 2–related factor 2 (NRF2) ameliorates P301S‐induced memory loss. (A) Experimental procedures of virus injection and behaviour tests. AAV‐P301S‐EGFP or AAV‐EGFP, and AAV‐NRF2‐mCherry or AAV‐mCherry were stereotaxically injected into the hippocampal CA3 region of 2‐month‐old C57BL/6J mice, and learning and memory were detected 4 weeks later. (B‐D) Efficiency of virus infection and P301S or NRF2 overexpression at CA3 were confirmed by fluorescence imaging or Western blotting at 4 weeks after virus infection. One‐way analysis of variance (ANOVA), *F*(3, 8) = 45.3, *p* < .01; *n* = 3. (E and F) Overexpressing NRF2 ameliorated P301S‐induced malondialdehyde (MDA) concentration and superoxide dismutase (SOD) activity in CA3 of C57BL/6J mice. One‐way ANOVA, [MDA] *F*(3, 8) = 23.13, *p* =.0016; [SOD] *F*(3, 8) = 19.78, *p* =.0038; *n* = 3. (G and H) Knockdown NRF2 decreased mice's staying time in centre zone of the box of open field test (OFT). One‐way ANOVA, *F*(3, 36) = 5.593, *p* = .0260; *n* = 10. (I and J) Overexpressing NRF2 increased mice's staying time in centre zone of the box of OFT. One‐way ANOVA, *F*(3, 36) = 3.488, *p* = .0255; *n* = 9–10 mice in each group. (K and L) Overexpressing NRF2 reversed P301S‐induced novel object recognition (NOR) impairments in C57BL/6J mice compared with the vehicle controls. Cartoons show paradigms of the NOR. One‐way ANOVA, *F*(3, 40) = 5.807, *p* = .0022; *n* = 10–11 mice in each group. (‐M and N) Overexpressing NRF2 ameliorated P301S‐induced spatial learning deficit in Morris water maze (MWM) test. Cartoons show paradigms of the MWM test. Two‐way ANOVA × days, *F*(3, 168) = 6.431, *p* = .0004; *n* = 9–10 mice in each group. (O–R) Overexpressing NRF2 ameliorated P301S‐induced spatial memory deficits in C57BL/6J mice shown by the increased platform crossing time (O), increased duration in the target quadrant (P) and decreased time to reach the platform location (latency) (Q) in water maze test detected at day 7 after removed the platform; the swimming speed was not changed (R). One‐way ANOVA, [platform crossing times] *F*(3, 36) = 5.795, *p* = .0024; [duration] *F*(3, 36) = 5.952, *p* = .0021; [latency] *F*(3, 36) = 5.018, *p* = .0024; *n* = 10 mice in each group. (S and T) Overexpressing NRF2 ameliorated P301S‐impaired fear conditioning test (FCT). One‐way ANOVA, *F*(3, 36) = 4.089, *p* = .0135; *n* = 10–11 mice in each group. * *p* < .05, ** *p* < .01 versus VEC + mCherry; # *p* < .05, ## *p* < .01 versus P301S + mCherry. Data were presented as mean ± SEM.

To investigate whether NRF2 reduction mediates P301S‐induced synaptic loss and disorders of memory and emotion, we injected AAV‐P301S or AAV‐NRF2 into the CA3 region of 2‐month‐old C57BL/6J mice (Figure 4A). After 4 weeks, the efficiency of the virus expression was detected by Western blotting and immunofluorescence images (Figure 4B,C). We detected a robust decrease in MDA and an increase in SOD in the P301S + NRF2 group (Figure 4E,F), suggesting that oxidative stress induced by P301S overexpressing was attenuated by NRF2. P301S‐induced anxiety‐like behaviour was measured by the OFT and EPM (Figures 4G–J, S8 and S9), as evidenced by more staying time in the centre zone of the OFT and less investigating time in the closed arm of the EPM than the P301S + mCherry group. In the NOR test, overexpressing NRF2 attenuated P301S^CA3^‐induced memory deficits shown by the increased time spent exploring the new object (Figures 4K,L and S9). Furthermore, the escape latency during 5‐day MWM training (Figure 4M,N) and in the probe test (Figure 4Q) was significantly decreased, whereas the platform crossing times (Figures 4O andS10) and staying time in the target quadrant (Figure 4P) were remarkably increased by NRF2 overexpression in P301S^CA3^ mice, without a significant difference in swimming speed among the groups (Figure 4R). In contextual FCT, mice with NRF2 overexpression exhibited more freezing time than the P301S^CA3^ group (Figure 4S,T), suggesting improved long‐term memory after NRF2 overexpression. These data demonstrate that NRF2 reduction is required for P301S^CA3^‐induced cognitive deficits and anxiety.

**FIGURE 5:**
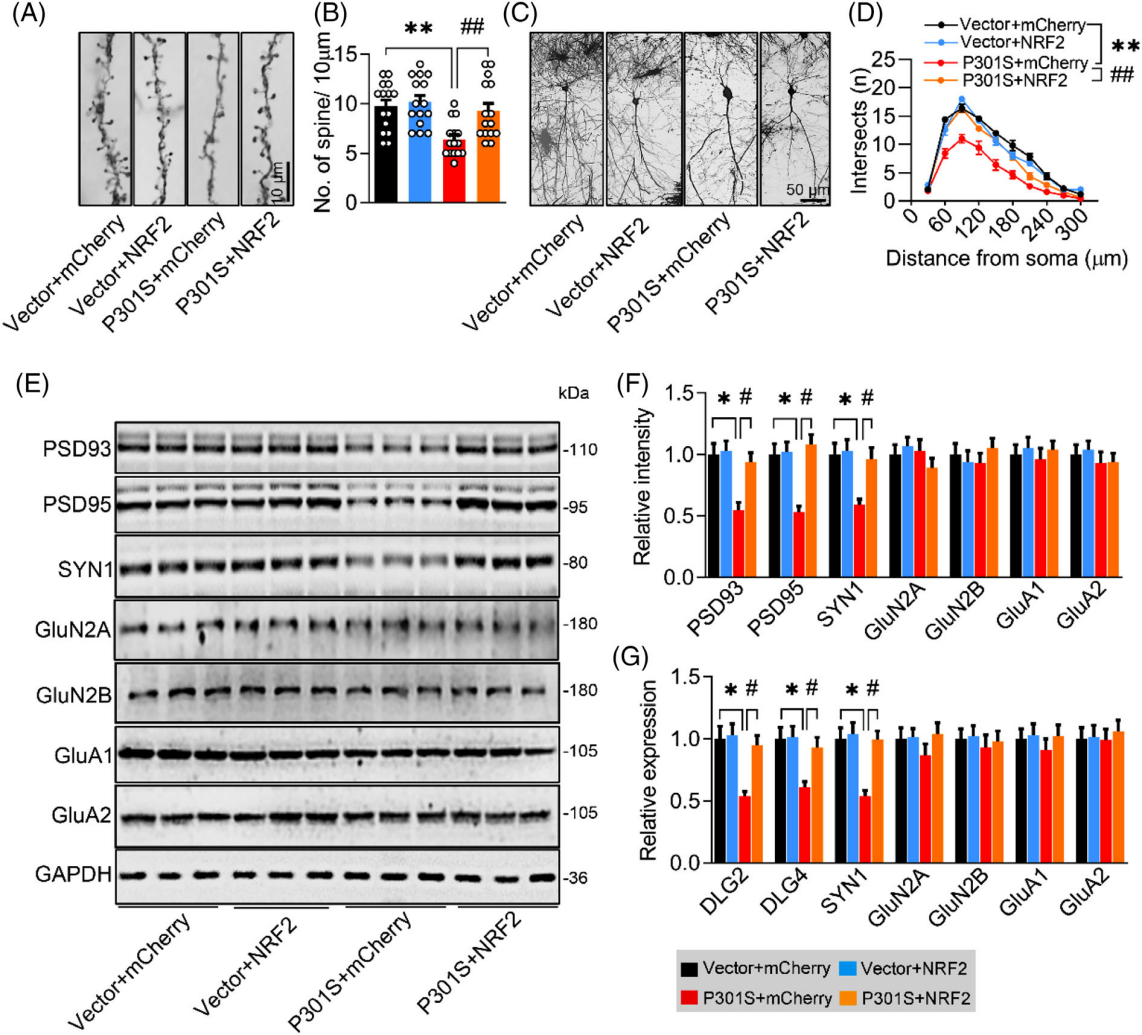
Overexpressing nuclear factor‐erythroid 2–related factor 2 (NRF2) ameliorates P301S‐induced synapse loss and synaptic protein reduction. (A and B) Overexpressing NRF2 reversed P301S‐induced synapse loss detected by Golgi‐cox staining. The representative images (A) and quantitative analysis (B) of spine. One‐way analysis of variance (ANOVA), *F*(3, 36) = 7.398, *p* = .0003; *n* = 15. (C and D) Overexpressing NRF2 reversed P301S‐impaired neural complexity in the hippocampus by Sholl analysis. The representative images (C) and quantitative analysis (D). Two‐way ANOVA × distance, *F*(3, 160) = 93.27, *p* < .0001. *p* = .001; *n* = 5. (E–G) Overexpressing NRF2 ameliorated P301S‐decreased protein (E and F) and mRNA levels (G) of synaptic proteins. One‐way ANOVA, (F) [PSD93] *F*(3, 8) = 6.233, *p* = .0173; [PSD95] *F*(3, 8) = 4.907, *p* = .0320; [SYN1] *F*(3, 8) = 7.694, *p* = .0096; *n* = 3. (G) [DLG2] *F*(3, 8) = 6.086, *p* = .0184; [DLG4] *F*(3, 8) = 7.303, *p* = .0112; [SYN1] *F*(3, 8) = 4.442, *p* = .0407; *n* = 3. * *p* < .05, ** *p* < .01 versus VEC + mCherry; # *p* < .05, ## *p* < .01 versus P301S + mCherry. Data were presented as mean ± SEM.

By Golgi‐cox staining, we found a significant increase in spine density (Figure 5A,B) and dendritic complexity (Figure 5C,D) in the P301S^CA3^‐NRF2 group. Compared with the P301S^CA3^ group, the protein expression of PSD93, PSD95 and SYN1 (Figure 5E,F) and mRNA levels of DLG2, DLG4 and SYN1 (Figure 5G) were also markedly increased in the P301S^CA3^‐NRF2 group. In P301S‐transfected N2a cells, we also observed that overexpressing NRF2 restored the levels of the synaptic proteins PSD93, PSD95 and SYN1 and the mRNA levels of DLG2, DLG4 and SYN1 (Figure S12).

Collectively, these data demonstrate that overexpressing NRF2 could efficiently reverse P301S‐induced synapse loss and disorders of memory and emotion.

### 3.4 Insufficient binding of NRF2 to specific ARE promoter elements decreases the transcription of synaptic genes in the presence of P301S

**FIGURE 6:**
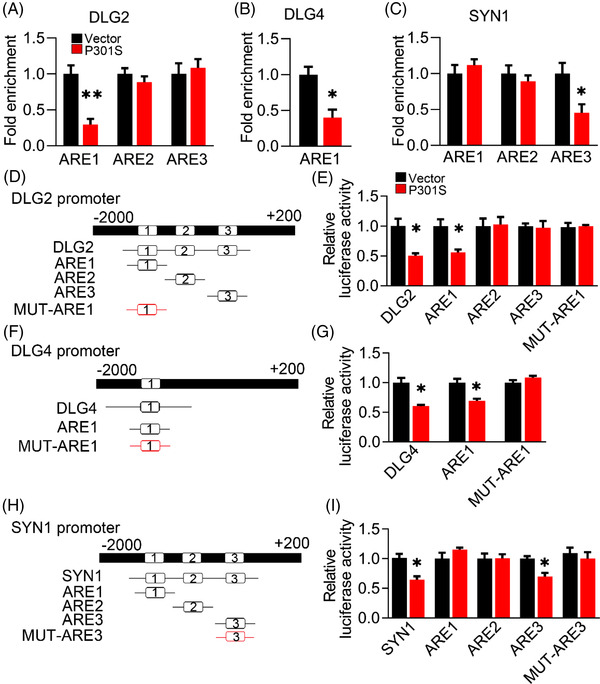
P301S inhibits binding of nuclear factor‐erythroid 2–related factor 2 (NRF2) to the promoter elements of several synaptic proteins. (A–C) Overexpressing P301S decreased the binding of NRF2 to the promoter regions of DLG2 (A), DLG4 (B) and SYN1 (C) genes in N2a cells, as measured by chromosome immunoprecipitation (ChIP). Unpaired *t*‐test, [DLG2] *t* = 4.888 df = 4, *p* = .0081; [DLG4] *t* = 3.899 df = 4, *p* = .0176; [SYN1] *t* = 2.842 df = 4, *p* = .0468; *n* = 3. (D, F and H) Diagrams show the predicted anti‐oxidative response element (ARE) for NRF2 binding in the promoter (−2000 ± 200 bp) of DLG2 (D), DLG4 (F) and SYN1 (H) genes. (E, G and I) The AREs or the mutated (MUT) AREs plasmids of DLG2 (E), DLG4 (G) and SYN1 (I) genes were co‐transfected with P301S or its empty vector (VEC) into HEK293 cells for 24 h, and then the luciferase activity was measured. (E) Unpaired *t*‐test, [DLG2] *t* = 3.89 df = 4, *p* = .0177; [ARE1] *t* = 3.603 df = 4, *p* = .0227. (G) Unpaired *t*‐test, [DLG4] *t* = 4.587 df = 4, *p* = .0101; [ARE1] *t* = 4.160 df = 4, *p* = .0141. (I) Unpaired *t*‐test, [SYN1] *t* = 4.279 df = 4, *p* = .0129; [ARE3] *t* = 4.118 df = 4, *p* = .0146; *n* = 3. *p < .05, **p < .01. Data were presented as mean ± SEM.

To address how NRF2 mediates P301S‐reduced expression of synaptic proteins, we applied the JASPAR platform (https://jaspar.genereg.net/) to analyse the binding element of NRF2. Given that the specific sequence of the ARE element for NRF2 is 5′‐TGACnnnGC‐3′ or 5′‐GCnnnnnnTCA‐3′, we found three conserved promoter elements of DLG2 and SYN1, and one conserved ARE promoter element of DLG4, for NRF2 binding. By the ChIP assay, we confirmed that overexpressing P301S significantly decreased the binding of NRF2 to the promoters of DLG2 (ARE1), DLG4 (ARE1) and SYN1 (ARE3) (Figure 6A–C).

Then, we constructed specific ARE promoter elements of synaptic proteins and conducted a DLR assay. By transfecting DLG2/DLG4/SYN1‐ARE‐plasmid constructs, we found significantly decreased luciferase activity in the P301S group compared with the control group (Figure 6D–I). However, this reduction was completely abolished when the mutated ARE plasmid was transfected (Figure 6D–I).

These data together suggest that insufficient binding of NRF2 to specific ARE promoter elements decreases the transcription of synaptic genes in the presence of P301S.

### 3.5 P301S acetylates KEAP1 to increase KEAP1 and promote NRF2 degradation via the ubiquitin–proteasome pathway

**FIGURE 7:**
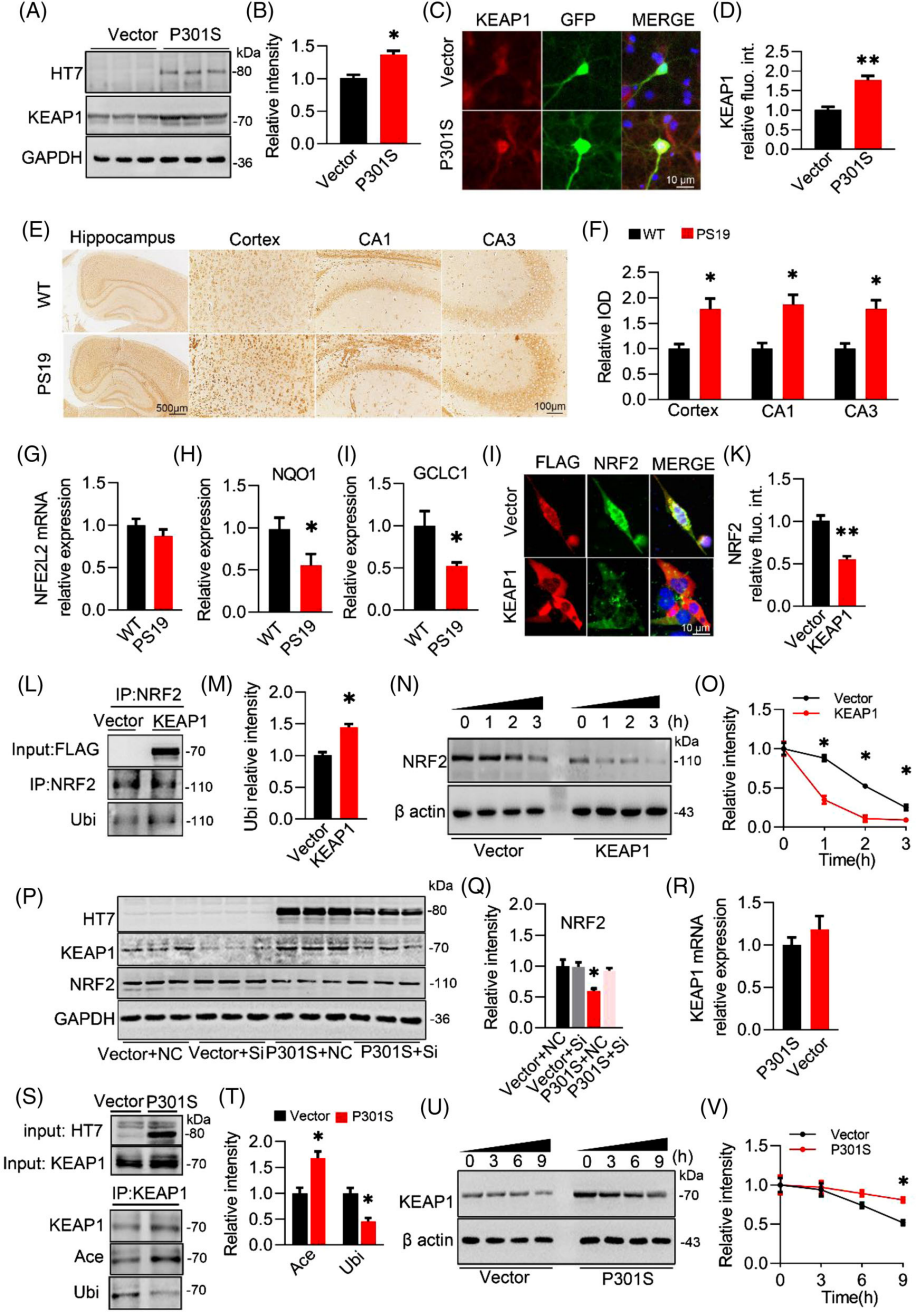
P301S acetylates Kelch‐like ECH‐associated protein 1 (KEAP1) and inhibits its ubiquitination and degradation. (A and B) Overexpressing P301S in N2a cells for 48 h increased KEAP1 expression. Unpaired *t*‐test, *t* = 4.445 df = 4, *p* = .0113; *n* = 3. (C and D) Overexpressing P301S in primary neurons by AAV‐P301S‐EGFP for 4 days increased the expression of KEAP1 detected by immunofluorescence staining. Unpaired *t*‐test, *t* = 5.735 df = 4, *p* = .0046; *n* = 3. (E and F) Increased KEAP1 was detected in the hippocampus and cortex of 8‐month‐old PS19 mice compared with S129 mice (E) and quantitative analysis (F). Unpaired *t*‐test, [CORTEX] *t* = 3.414 df = 4, *p* = .0269; [CA1] *t* = 3.963 df = 4, *p* = .0166; [CA3] *t* = 3.944 df = 4, *p* = .0169; *n* = 3. (G–I) mRNA level of NFE2L2, NQO1 and GLCL in CA3 of PS19 and WT mice. Unpaired *t*‐test, [NQO1] *t* = 5.322 df = 4, *p* = .0311; [GCLC] *t* = 5.382 df = 4, *p* = .0252; *n* = 3 (J and K) Overexpressing KEAP (FLAG‐label) in N2a cells decreased protein level of nuclear factor‐erythroid 2–related factor 2 (NRF2) measured by immunofluorescence staining. Unpaired *t*‐test, [NRF2] *t* = 6.232 df = 4, *p* = .0034; *n* = 3. (L and M) An increased ubiquitination (Ubi) of NRF2 was detected after overexpressing KEAP1 in primary hippocampal neurons examined by immunoprecipitation (IP) and Western blotting. Unpaired *t*‐test, [Ubi/NRF2] *t* = 4.537 df = 4, *p* = .0209; *n* = 3. (N and O) Overexpressing KEAP1 promoted proteolysis of NRF2. KEAP1 plasmid was transfected into N2a cells for 48 h, then cycloheximide (100 μM) were added into medium to inhibit ex vivo protein translation, and cells were harvested at 0, 1, 2 and 3 h after treatment and protein level of NRf2 was assayed by Western blotting using β actin as loading control. Two‐way analysis of variance (ANOVA) × *h*, *F*(1, 16) = 6.754, *p* = .0246; *n* = 3. (P and Q) Knockdown KEAP1 by siRNA ameliorated P301S‐induced decreased NRF2. One‐way ANOVA, *F*(3, 8) = 10.423, *p* = .0031; *n* = 3. (R) mRNA of KEAP1 in N2a cells after overexpressing P301S. (S and T) An increased acetylation (Ace) with decreased ubiquitination (Ubi) of KEAP1 was detected after overexpressing P301S in primary cultured neurons examined by IP and Western blotting presented by a ratio of Ubi or Ace to KEAP1 Ubiquitination level (Ubi) and acetylation level (Ace) of KEAP1. Unpaired *t*‐test, [Ace/KEAP1] *t* = 3.993 df = 4, *p* = .0162; [Ubi/KEAP1] *t* = 4.381 df = 4, *p* = .0119; *n* = 3. (U and V) Overexpressing P301S inhibited proteolysis of KEAP1. P301S plasmid was transfected into N2a cells for 48 h, then cycloheximide (100 μM) were added into medium to inhibit ex vivo protein translation, and cells were harvested at 0, 3, 6 and 9 h after treatment and protein level of KEAP1 was assayed by Western blotting using β actin as loading control. Two‐way ANOVA × *h*, *F*(1, 16) = 5.401, *p* = .0336; *n* = 3.*p < .05, **p < .01. Data were presented as mean ± SEM.

To investigate the molecular mechanism underlying the P301S‐induced NRF2 reduction, we first examined the expression of KEAP1, a well‐known negative repressor of NRF2. A significantly increased KEAP1 protein level was shown by Western blotting after transient transfection of the P301S plasmid into N2a cells (Figure 7A,B). Interestingly, the increase of KEAP1 was specific in the P301S group, not in the wild‐type hTau and P301L hTau groups (Figure S13). Abundant KEAP1 protein was also observed within the neuronal cell bodies and neurites in P301S‐infected primary neurons (Figure 7C,D), and in the brain sections of cortex, hippocampal CA1 and CA3 subsets of PS19 mice (Figure 7E,F), which further confirmed the upregulation of KEAP1 protein by P301S accumulation.

Next, we measured NRF2 protein in the presence of P301S and KEAP1. In PS19 mice, along with the increase in KEAP1, the protein level, but not the mRNA level, of NRF2 was significantly decreased in the hippocampal CA3 compared with the WT group along with decreased NQO1 and GCLC1 (two downstream target genes of NRF2) (Figure 7G–I), indicating that the post‐translation modification may be involved in the reduction of NRF2 by KEAP1. Next, we overexpressed KEAP1 in N2a cells and measured the alterations in ubiquitinated NRF2. By immunofluorescence staining, a remarkable reduction in NRF2 within the cell bodies and nuclear fractions was obviously detectable in KEAP1‐overexpressing N2a cells (Figure 7K,J). Simultaneously, the ubiquitin level of NRF2 was significantly increased in the KEAP1 group compared with the control group (Figure 7L,M). In the presence of CHX, the degradation rate of NRF2 in the KEAP1 group was much faster than that in the control groups (Figure 7N,O). When KEAP1 was knocked down in the P301S group, the reduction in NRF2 protein levels was completely abolished and reached the level of the control group (Figure 7P,Q). These data indicate that KEAP1 plays an essential role in P301S‐decreased NRF2 proteins via the ubiquitin–proteasome pathway.

Then, we aimed to investigate how P301S increased the protein level of KEAP1. By qPCR, no significant difference in *KEAP1* mRNA was detected between the P301S and vector groups (Figure 7R), indicating the contribution of the post‐translation modification to P301S‐increased KEAP1. Given that Tau could upregulate protein levels via its acetyltransferase activity,^31^ we asked whether the P301S‐induced increase in KEAP1 might be attributed to the acetyltransferase activity of P301S. Compared with the wild‐type hTau and P301L hTau, a higher combination affinity to KEAP1 proteins was observed in P301S‐transfected N2a cells (Figure S14), indicating the vulnerability of KEAP1 to P301S accumulation. Next, we enriched KEAP1 proteins in the cell extracts by IP using a KEAP1 antibody and then examined the acetylation level of KEAP1 by using a pan‐acetylated lysine antibody (ACE). Compared with the control, overexpressing P301S (detected by HT7) significantly increased the acetylation level of KEAP1 (input), accompanied by a decreased ubiquitin level (using pan‐ubiquitinated lysine antibody, Ubi) (Figure 7S,T). Meanwhile, impaired proteolysis of KEAP1 was detected (Figure 7U,V) after introducing P301S.

Collectively, our data demonstrate that P301S acetylates KEAP1 to increase KEAP1 proteins and promotes NRF2 degradation via ubiquitin–proteasome pathway.

### 3.6 Blocking P301S–KEAP1 interaction efficiently ameliorates P301S‐induced synapse loss and cognitive dysfunction

Next, we investigated whether blocking the P301S–KEAP1 interaction could ameliorate P301S‐induced synapse loss and memory deficits. By using GPS‐PAIL, K84 and K312 of KEAP1 were predicted to be the targets of tau. Next, we constructed mutated KEAP1 and performed co‐immunoprecipitation (CoIP) to investigate which site is critical for the P301S–KEAP1 interaction. Compared with WT, KEAP1 mutation at K312, not K84, significantly decreased its association with P301S (Figure S16a–d). Furthermore, we performed a proximity ligation assay (PLA) experiment in N2a cells transfected with P301S and distinct KEAP1. Compared with the vector, PLA dots were observed in N2a cells overexpressing KEAP‐WT and P301S, indicating an association between P301A and KEAP. However, the intensity of PLA dots significantly decreased when KEAP1 was mutated at K312. No difference in PLA dots was detected between the KEAP‐WT and KEAP‐K84R groups (Figure S16E,F). These data demonstrate that K312 of KEAP1 is essential for the P301S–KEAP1 association.

Then, we designed two peptides to target K84 (Peptide P1: CDVTLQVKYQDAPAA) and K132 (P2: FEELTLHKPTQAVPC). The CCK8 test on N2a cells showed a significant reduction in cell viability at concentrations of 400‐μm P1 and 200‐μm P2 (Figure S15a,c), indicating that the relatively safe doses of P1 and P2 were 25–200 and 25–100 μm, respectively. Next, we performed IP to evaluate the blocking efficiency of P1 and P2. P1 and P2 were added to the N2a cells. After 48 h, we harvested the cells and detected a significant decrease in the P301S–KEAP1 interaction in the P2‐treated group, but not P1‐treated group (Figure S15a–d).

Moreover, only P2 at 100 μm robustly decreased the acetylation level of KEAP1 proteins in the presence of P301S (Figure S16g,h). These data indicate that competitive blockade of K312 of KEAP1 by P2 could efficiently decrease the P301S–KEAP1 interaction and P301S‐acetylated KEAP1.

**FIGURE 8:**
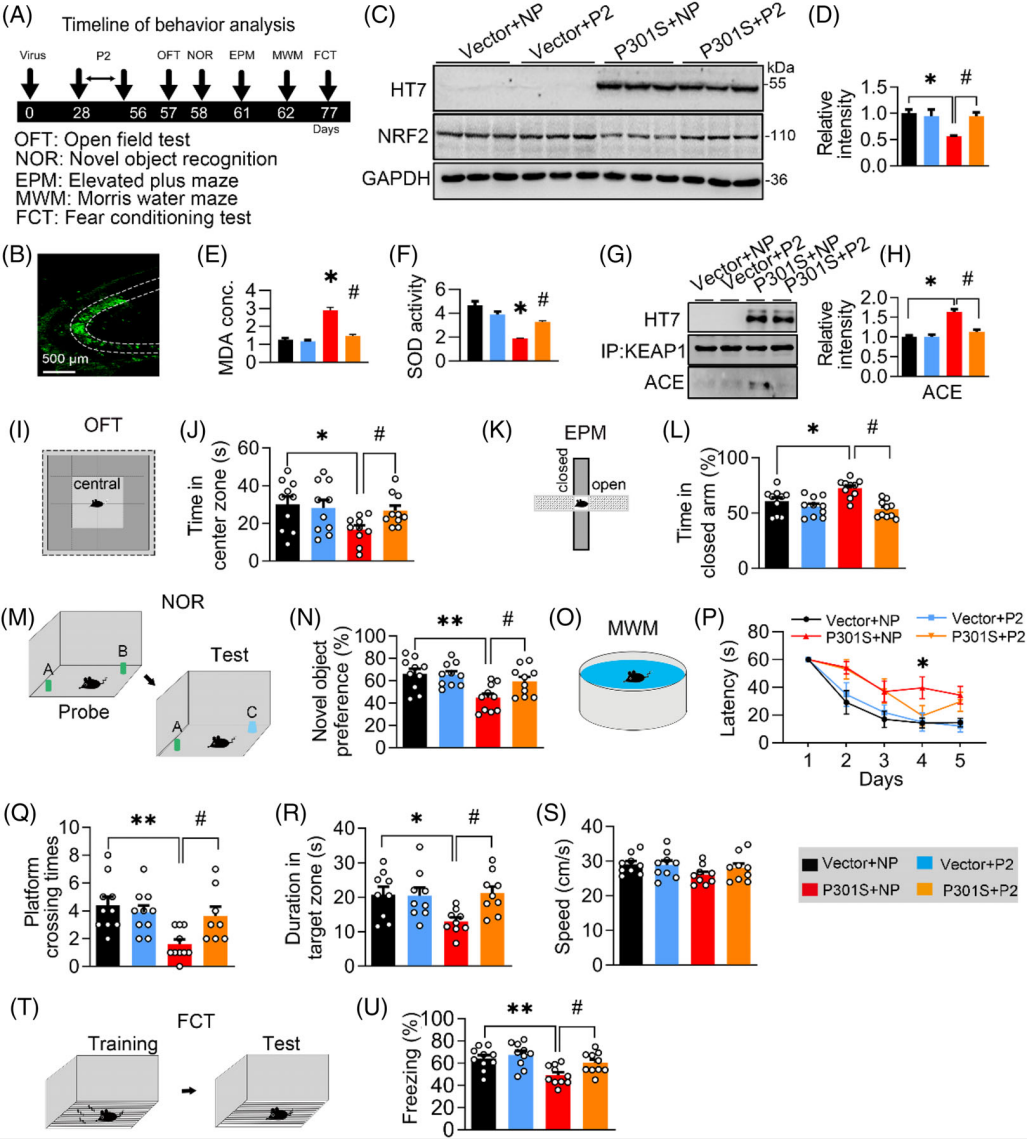
Blocking P301S–Kelch‐like ECH‐associated protein 1 (KEAP1) interaction by a custom‐designed peptide ameliorates P301S‐induced memory loss. (A) Experimental procedures of virus injection, peptide administration and behaviour test. AAV‐P301S‐EGFP or AAV‐EGFP was stereotaxically injected into the hippocampal CA3 region of 2‐month‐old C57BL/6J mice for 4 weeks. Then P2 or negative peptide (NP) was administrated via intracerebroventricular injection (every 3 days for 4 weeks) after virus injection. The capacity of learning and memory was measured 1 day after last administration. (B) Efficiency of virus infection at CA3 was confirmed by GFP fluorescence. (C and D) Protein levels of P301S or nuclear factor‐erythroid 2–related factor 2 (NRF2) were confirmed by Western blotting and quantitative analysis. One‐way analysis of variance (ANOVA), *F*(3, 8) = 6.173, *p* = .0179; *n* = 3. (E and F) P2 ameliorated P301S‐induced malondialdehyde (MDA) concentration and superoxide dismutase (SOD) activity in CA3 of C57BL/6J mice. One‐way ANOVA, [MDA] *F*(3, 8) = 18.94, *p* =.0032; [SOD] *F*(3, 8) = 17.86, *p* =.0038; *n* = 3. (G and H) P2 ameliorated P301S‐induced acetylation of KEAP1 in CA3 of C57BL/6J. One‐way ANOVA, [ACE] *F*(3, 8) = 24.51, *p* =.0018; *n* = 3. (I and J) P2 ameliorated mice's staying time in centre zone of the box of open field test (OFT). One‐way ANOVA, *F*(3, 36) = 3.680, *p* = .0207; *n* = 10 mice in each group. (K and L) P2 reversed mice's staying time in closed arm of frame in elevated plus maze (EPM) test. One‐way ANOVA, *F*(3, 36) = 3.540, *p* = .0085; *n* = 10 mice in each group. (M and N) P2 reversed P301S‐impaired novel object recognition (NOR). Cartoons show paradigms of the NOR. One‐way ANOVA, *F*(3, 36) = 6.338, *p* = .0015; *n* = 10 mice in each group. (O and P) P2 ameliorated P301S‐induced spatial learning deficit in Morris water maze (MWM) test (O). Cartoons show paradigms of the MWM test (P). Two‐way ANOVA × days, *F*(3, 155) = 11.51, *p* < .0001; *n* = 10 mice in each group. (Q‐S) P2 ameliorated P301S‐induced spatial memory deficits in C57BL/6J mice shown by the increased platform crossing times (Q), duration in the target quadrant (duration) (R) in water maze test detected at day 7 after removing the platform without affecting swimming speed (S). One‐way ANOVA, [platform crossing times] *F*(3, 34) = 5.727, *p* = .0028; [duration] *F*(3, 32) = 3.933, *p* = .0170; *n* = 8–10 mice in each group. (T and U) P2 reversed P301S‐impaired fear conditioning test (FCT) compared with the negative peptide. Cartoons show paradigms of the behavioural test of FCT. One‐way ANOVA, *F*(3, 36) = 6.764, *p* = .0010; *n* = 10 mice in each group. *p < .05, **p < .01 versus VEC + NP; #*p* < .05 versus P301S + NP. Data were presented as mean ± SEM.

Then, we infused P2 into the lateral cerebral ventricle of P301S^CA3^ mice (once every 3 days for 4 weeks) (Figure 8A). Consistently, P2 administration significantly increased NRF2 (Figure 8C,D), decreased MDA concentration (Figure 8E), and KEAP1 acetylation (Figure 8G,H) and increased SOD activity (Figure 8F). Behaviourally, P301S‐induced anxiety was completely reversed by P2 as measured by the OFT and EPM (Figures 8I–L, S17 and S18), as evidenced by increased staying time in the central zone of the OFT and decreased exploring time in the closed arm of the EPM. For cognitive capacity, P2 administration significantly raised the novel object preference in the NOR test (Figures 8M and S19). During MWM training, P301S^CA3^ mice with P2 treatment spent less time reaching the platform than the P301S group (Figure 8K,L), indicating an improvement in spatial learning ability. During the probe test, more platform crossing times (Figures 8Q and S20) and duration time in the target quadrant (Figure 8R) were observed in P301S^CA3^ mice with P2 treatment than in P301S^CA3^ mice, without a significant difference in swimming speed among the groups (Figure 8S). In contextual FCT, P2 mice exhibited longer freezing times than P301S^CA3^ mice, suggesting improved long‐term memory after P2 supplementation (Figure 8T,U). These data suggest that blocking the P301S–KEAP1 interaction with P2 could efficiently ameliorate P301S‐induced cognitive deficits and anxiety.

**FIGURE 9:**
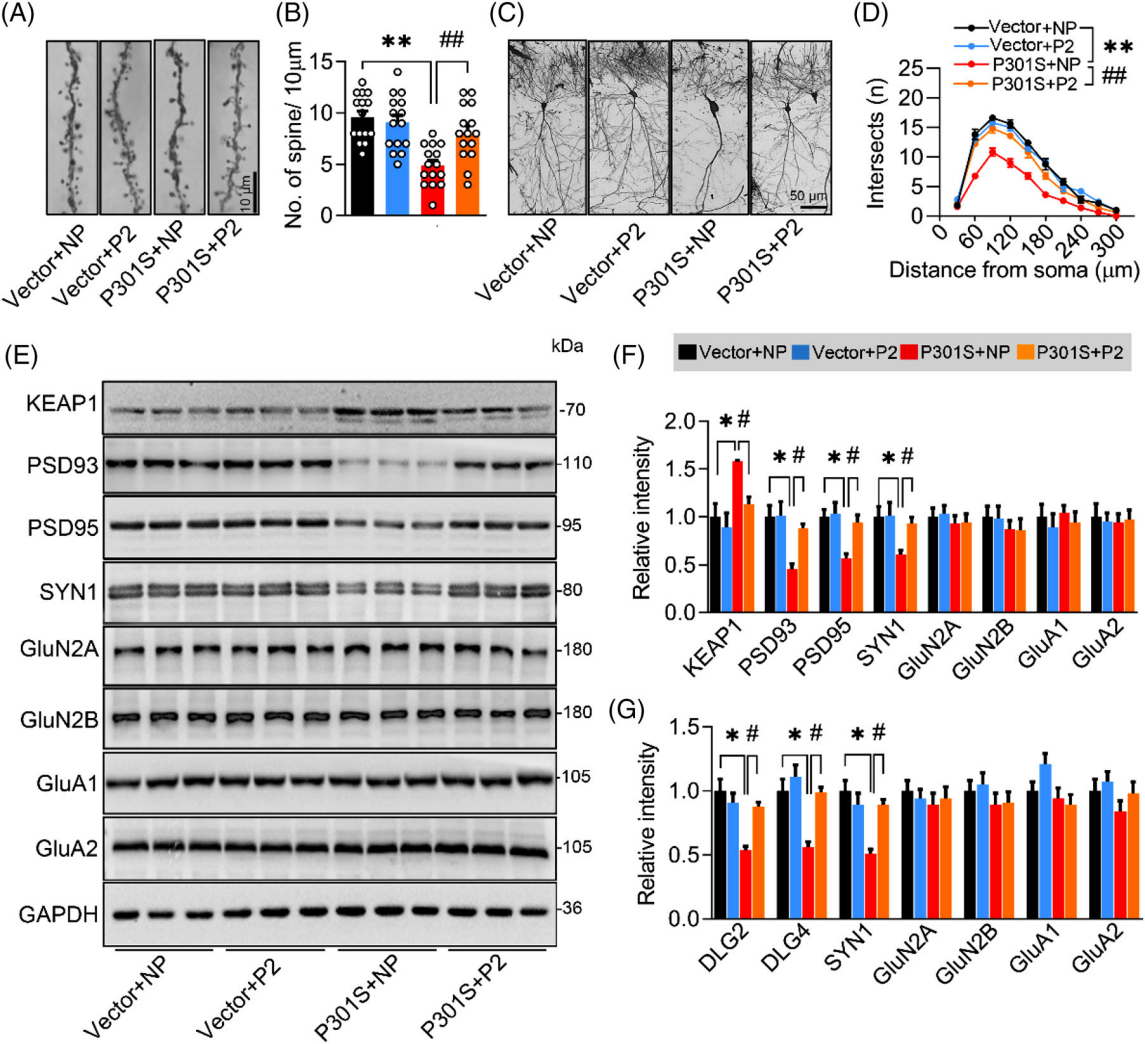
Blocking P301S–Kelch‐like ECH‐associated protein 1 (KEAP1) interaction by a custom‐designed peptide attenuates P301S‐induced synapse loss and synaptic protein reduction. (A and B) P2 reversed P301S‐decreased spine density detected by Golgi‐cox staining. One‐way analysis of variance (ANOVA), *F*(3, 56) = 11.54, *p* < .0001; *n* = 15. (C and D) P2 reversed P301S‐impaired neural complexity in the hippocampus by Sholl analysis. Two‐way ANOVA × distance, *F*(3, 160) = 114.7, *p* < .0001; *n* = 5. (E–G) P2 ameliorated P301S‐decreased protein (E and F) and mRNA (G) levels of synaptic proteins. One‐way ANOVA, for panel (F) [KEAP1] *F*(3, 8) = 7.632, *p* = .0198; [PSD93] *F*(3, 8) = 9.645, *p* = .0046; [PSD95] *F*(3, 8) = 9.828, *p* = .0179; [SYN1] *F*(3, 8) = 8.137, *p* = .0082, *n* = 3. For panel (G) [DLG2] *F*(3, 8) = 5.537, *p* = .0236; [DLG4] *F*(3, 8) = 6.705, *p* = .0142; [SYN1] *F*(3, 8) = 4.938, *p* = .0315; *n* = 3. *p < .05, **p < .01 versus VEC + NP; #*p* < .05, ##*p* < .01 versus P301S + NP. Data were presented as mean ± SEM.

Next, we measured synapse density and the expression of synaptic proteins in P301S^CA3^ mice after P2 infusion. By Golgi‐cox staining, we found that the reduction in spine density (Figure 9A,B) and dendritic complexity (Figure 9C,D) in P301S^CA3^ mice was remarkably reversed by P2 treatment. Moreover, the P301S‐induced decreases in PSD93, PSD95 and SYN1 proteins (Figure 9E,F) and DLG2, DLG4 and SYN1 mRNA levels (Figure 9G) were also restored to the control levels after 4‐week P2 administration.

As the NRF2 protein level was elevated simultaneously with the KEAP1 decrease after blocking the P301S–KEAP1 interaction by P2, we next aimed to confirm whether NRF2 mediates the beneficial effects of P2. After knocking down NRF2 in P301S‐overexpressing N2a cells treated with P2, Western blotting was conducted to measure the synaptic proteins. The improvement of PSD93, PSD95 and SYN1 by P2 was significantly attenuated by NRF2 knockdown (Figure S21a,b), indicating that NRF2, a downstream target of KEAP1, mediates the beneficial effects of P2.

Together, these data demonstrate that blocking the P301S–KEAP1 interaction can modify the KEAP1‐NRF2‐ARE pathway and efficiently ameliorate P301S‐induced synapse loss, thereby improving cognitive capacity and emotion status.

## 4 DISCUSSION

Synapse loss is an early event in the process of tauopathies.^32^, ^33^ However, whether and how the accumulated Tau triggers synaptic dysfunction and memory disorder are still elusive. In the present study, we exogenously overexpressed P301S in cells and rodents to mimic Tau accumulation in FTDP‐17 patients and found that overexpressing P301S‐induced oxidative stress, synapse loss and memory impairments with significantly decreased NRF2 and increased KEAP1. Mechanistically, P301S can acetylate KEAP1 to inhibit ubiquitination‐associated proteolysis. As an inhibitor, the elevated KEAP1 significantly suppressed the protein level of NRF2 in response to P301S accumulation. Interestingly, we found that NRF2 could bind to specific ARE promoter elements and thus activate the transcription of synaptic proteins. Overexpressing NRF2 increased the expression of synaptic proteins with improved memory, whereas the knockdown of NRF2 mimicked P301S‐induced synaptic and cognitive deficits. Importantly, we found that blocking the P301S–KEAP1 interaction at K312 efficiently ameliorated P301S‐induced synapse loss and cognitive dysfunction. Our results not only uncover a novel function of the KEAP1/NRF2/ARE pathway in regulating synaptic protein expression but also highlight an essential role of KEAP1/NRF2/ARE pathway in P301S‐induced synaptic toxicity and memory loss. Moreover, our custom‐designed peptide may have the potential to rescue the KEAP1/NRF2/ARE pathway and thus arrest tauopathies.

NRF2 is a master regulator in the antioxidant response and detoxification.^34^, ^35^ As a transcriptional regulator, NRF2 can bind to the ARE sequence and activate or inactivate gene expression. For example, NRF2 represses the expression of β‐site APP‐cleaving enzyme (BACE1) and BACE1‐mRNA‐stabilizing antisense RNA by binding to AREs in their promoters, consequently preventing the Aβ pathogenic process in AD.^36^ Both genetic and pharmacological inductions of NRF2 could suppress inflammatory cytokine genes and glutathione synthesis genes, thereby attenuating oxidative stress and improving cognitive function in an AD mouse model.^24^ In addition to anti‐oxidative stress, NRF2 in astrocytes exerts beneficial effects on cerebral hypoperfusion via repressing the expression of complement components.^23^ However, the function of NRF2 remains unknown. In the present study, we observed that knocking down NRF2 significantly decreased the expression of synaptic proteins, such as PSD93, PSD95 and SYN1. Within the promoter region of the DLG2, DLG4 and SYN1 genes, ARE elements for NRF2 binding were identified by bioinformatic analysis. Using a ChIP assay, we found that overexpressing P301S significantly decreased the binding of NRF2 to the promoters of DLG2 (ARE1), DLG4 (ARE1) and SYN1 (ARE3). Consistently, our luciferase activity assay data showed a decrease in luciferase activity in the P301S group compared with the control group, and this suppression was completely abolished when the mutated ARE plasmid constructs were transfected. We also found that overexpressing NRF2 in vivo efficiently ameliorated the P301S‐induced reduction in synaptic proteins and synapse loss. However, these beneficial effects of NRF2 on synapse in the present study are not consistent with the findings from Bell et al.^37^ Bell et al. performed experiments on primary cortical neurons, whereas we focused on primary hippocampal neurons. Although there are no reports revealing the difference between primary cortical neurons and primary hippocampal neurons, their distinct nature as seen in vivo^38^, ^39^ may probably contribute to the inconsistency of different NRF2 on synapse. Additionally, mature neurons in vivo as investigated in the present study may not be the same as developing neurons in vitro as studied by Bell et al., which may result in the discrepancy of NRF2 on synapses. Therefore, our results not only uncover a novel function of NRF2 in regulating the expression of specific synaptic proteins in mature hippocampal neurons but also indicate a crucial role of NRF2 in mediating P301S‐induced synaptic dysfunctions.

KEAP1 was the first identified and a well‐recognized NRF2 suppressor.^40^, ^41^ In the cytoplasm, there is a tight interaction between NRF2 and KEAP1.^42^ The N‐terminus of KEAP1 links with Cullin‐3 to form a ligase complex with E3 ubiquitin, which ubiquitinates NRF2, leading to constitutive degradation of NRF2 through the ubiquitin–proteasome system.^43^ In the present study, we detected increased KEAP1 along with decreased NRF2 in the presence of P301S accumulation. Overexpression of KEAP1 significantly raised the ubiquitinated NRF2 proteins and increased NRF2 degradation, thereby reducing the NRF2 protein level. Our findings are not consistent with observations from primary cortical neurons, which revealed that NRF2 was only epigenetically repressed by the Class I/II histone deacetylase, not by KEAP1 during the development of primary cortical neurons.^37^ As NRF2 was significantly decreased in all hippocampal subregions, especially CA3, except the cortex of PS19 mice, we speculate that the discrepancy between ours and a previous report may be attributed to the different cell/neuron types (i.e. hippocampal neurons vs. cortical neurons) and distinct statuses (i.e. mature vs. developing). We uncovered an essential role of KEAP1 in NRF2 inhibition during P301S accumulation; however, the interaction site(s) between KEAP1 and NRF2 should deserve to be investigated in the future. As P301S‐increased KEAP1 was incapable of decreasing NRF2 proteins as exogenous NRF2 was introduced, the underlying mechanism, such as P301S‐NRF2 crosstalk, needs to be further studied.

Increasing evidences reveal a positive correlation between hTau accumulation and cognitive dysfunction.^44^, ^45^ For example, non‐phosphorylated hTau was enriched in synapses along with synapse loss and memory deficits in P301S transgenic mice.^46^ Comparing RNA profiling between 2‐ and 12‐month‐old P301S mice, the majority of the differentially expressed genes were associated with synaptic signalling.^47^ We previously reported that overexpressing wild‐type hTau or mutated P301L hTau could decrease the expression of *N*‐methyl‐d‐aspartate (NMDA) receptors via activating the Janus kinase/signal transducer and activator of transcription pathway.^27^, ^48^, ^49^ In the present study, we detected a reduction in synaptic proteins and memory loss after P301S overexpression. Interestingly, we detected reductions in PSD93, PSD95 and SYN1 but not NMDARs proteins after P301S overexpression, indicating the particularity of P301S toxicity on the synapses. Furthermore, we also found that the KEAP1/NRF2 pathway mediated the P301S‐induced downregulation of synaptic proteins, that is, PSD93, PSD95 and SYN1, as evidenced by the recovery of PSD93, PSD95 and SYN1 levels after NRF2 overexpression and the P301S–KEAP1 interaction blockade. By CoIP and PLA, we identified K312 of KEAP1 for the P301S–KEAP1 interaction and P301S‐directed acetylation. As described by the published literature, Tau R2/R3 (R2: DLSNVVSKGGS and R3: DLSKVTSKCGS) motifs are responsible for its acetyltransferase activity,^50^ and it is speculated that P301S‐hTau interacts with KEAP1 through its R2/R3 motif and promotes an acetylation of KEAP1 at K312. Compared with P301S, P301L and wild‐type hTau exerted weak interaction affinity to KEAP1, which may be ascribed to P301S having a more favourable conformation to form fibrillars.^51^ Therefore, in addition to the distinct spatial conformation, the difference in interaction site(s) among P301S, P301L and wild‐type hTau to KEAP1 may deserve further investigation.

In summary, we found that the KEAP1/NRF2 pathway mediates P301S‐induced synaptic protein loss and memory dysfunctions. Blocking the P301S–KEAP1 interaction or overexpressing NRF2 can increase the expression of synaptic proteins and improve cognitive functions in P301S^CA3^ mice. Therefore, targeting the KEAP1/NRF2 pathway, such as by competitively blocking K312 of KEAP1, downregulating KEAP1 and upregulating/activating of NRF2, may potentially antagonize P301S toxicity in future translational studies.

## CONFLICT OF INTEREST

The authors report no biomedical financial interests or potential conflicts of interest.

## Supporting information



Supp information
