# Supplementary material for: P301S‐hTau acetylates KEAP1 to trigger synaptic toxicity via inhibiting NRF2/ARE pathway: A novel mechanism underlying hTau‐induced synaptic toxicities
Source: Clin Transl Med. 2022 Aug 2;12(8):e1003. doi: 10.1002/ctm2.1003 (PMC9345400; doi:10.1002/ctm2.1003)
Supplement: Supplementary file 1 — Supp information [file CTM2-12-e1003-s001.docx]

**P301S-hTau acetylates KEAP1 to trigger synaptic toxicity via inhibiting NRF2/ARE pathway: a novel mechanism underlying hTau-induced synaptic toxicities**

Jia-Zhao Xie, Yao Zhang, Shi-Hong Li, Hui Wei, Hui-Ling Yu, Qiu-Zhi Zhou, Lin-Yu Wei, Dan Ke, Qun Wang, Ying Yang, Jian-Zhi Wang

Supporting information

**Supplementary methods**

**1.1 Immunofluorescence and immunohistochemistry staining**

For immunofluorescence (IF), cells cultures were firstly plated onto glass coverslips. After transfection with plasmids for 24 h, cells were fixed with 4% paraformaldehyde at RT for 30 min. After being blocked with 3% BSA in PBS with for 30 min at RT, cells were incubated with primary antibodies overnight at 4°C overnight. After being rinsed, the coverslips were incubated with secondary antibody solution for 1 h at RT. Nuclear staining was accomplished by adding DAPI fluorescence dye. The coverslips were rinsed with PBS, and mounted onto glass slides with 50% glycerin in PBS. Fluorescence of cells was analyzed by VS120 microscope (Olympus, Japan), intensity of fluorescence was analyzed by Image J (NIH, USA).

For DHE staining, cells cultures were firstly plated onto glass coverslips. After transfection with indicated plasmids for 24 h, DHE (1 μM) was added into cell medium and incubated at 37 °C for 0.5 h. After being rinsed, nuclear staining was accomplished by adding DAPI fluorescence dye. The coverslips were rinsed with PBS, and mounted onto glass slides with 50% glycerin in PBS. Fluorescence of DHE was analyzed by VS120 microscope (Olympus, Japan), intensity of fluorescence was analyzed by Image J (NIH, USA).

For immunohistochemistry (IHC), mouse brain sections were incubated in 0.1% triton in PBS for 30 min. After being rinsed, mouse sections were blocked with 3% BSA at room temperature (RT) for 30 min. Then specific primary antibody was added into section and incubated overnight at 4°C. 12-16 h later, all sections were washed by PBS and incubated with peroxidase-conjugated anti-mouse or rabbit IgG at RT for 1 h and then reacted with a mixture of 0.05% 3,30-diaminobenzidine (DAB) containing 0.01% H_2_O_2_ for color reactions. For quantitative assessment, more than 5 fields were randomly selected and analyzed with ImageJ (NIH, USA).

**1.2 Western blotting and immunoprecipitation**

After virus injection in hippocampus or plasmids transfection into N2a cell, proteins were extracted and examined according to previous reported study. For acetylation of KEAP after immunoprecipitation, cultured N2a cells were pretreated with MG132 (10 μM) for 1 h, and then lysed with RIPA lysis buffer (1 mM PMSF) on ice for 10 min. Supernatants were collected and incubated with A+G agarose at 4 °C for 30 min, and then centrifuged at 8,000 × g, 10 min at 4 °C. Supernatants were incubated with specific primary antibodies and protein A+G agarose at 4 °C for 12-16 h. After centrifugation at 2000 × g for 5 min, the agarose beads were washed with pre-cold PBS three times, and resuspended in 40 μL SDS buffer (10% glycerol, 2% SDS, 100 mM dithiothreitol and 0.25% bromophenol blue), and then boiled at 95 °C for 10 min. After centrifugation at 10000 g for 5 min, supernatants were analyzed by Western blotting. For KEAP1’s degradation assay, N2a cells were treated with cycloheximide (CHX, 100 μM) after 48 h of transfection with P301S-hTau, then cells were harvested every 3 h until 9 h.

Tissues or cultured cells were harvested on ice, and homogenized with RIPA lysis buffer (1 mM PMSF), and then centrifuged at 12,000 × g at 4°C for 10 min. The supernatant was collected and measured through BCA assays. Protein from each sample were loaded on 8-15% SDS-PAGE gels, and then transferred onto 0.45 μm polyvinylidene fluoride membranes (PVDF, Merck Millipore, USA). The membranes were blocked with 5% non-fat milk at RT, and incubated with antibodies overnight at 4°C, and then HRP-conjugated secondary antibodies for 1 h at RT. The blots were developed by ECL substrate in luminometer (ChemiScope 6000, Clinx, China), and quantified using ImageJ software (NIH, USA).

**1.3 Reverse transcription and quantitative polymerase chain reaction (qPCR)**

Total RNA was isolated from cells or hippocampus using TRIzol Reagent according to the manufacturer’s manual. Complementary DNA (cDNA) was synthesize using Hifair® Ⅱ 1st Strand cDNA Synthesis Kit and qPCR assays were performed using SYBR mix. The PCR system contains 0.5 μM primers (forward and reverse), 5 μl SYBR Green master mixes, and 1 μl cDNA. The mix samples were assayed on machine ABI7500 (Applied Biosystem, USA). The targeted gene’s expression was normalized according to the housekeeping gene ACTB. Primer for qPCR were listed in supplementary Table S2.

**1.4 Behavioral tests**

OFT: The mice entered the arena from the same location and were granted 5-min exploring. After finishing, the arena was cleaned with 75% ethanol. The behavior was recorded and analyzed by a video camera (Techman, Chengdu, China). The time mice stayed in center zone was recorded.

NOR: Mice were habituated to the arena for 5 min without object one day prior to the test. At training day, all mice were placed into the arena, and explored two different objects termed as A and B for 5 min. After training period, the objects and arena were cleaned with 75% ethanol. At testing day, object B was substituted with new object C, and the mice were allowed to explore both objects for 5 min. The behavior was recorded and analyzed by a video camera (Techman, Chengdu, China). The time mice touched object A was marked as TA, the time mice touched object C was marked as TC. The recognition index was calculated by TC/ (TA + TC).

EPM: Mice were first placed at the intersection of the closed and open arms (Techman, Chengdu, China), and granted 5-min exploring. After finishing, the closed and open arms were cleaned with 75% ethanol. The time mice stayed in closed or open arm was recorded.

MWM: All mice were trained in a maze for 5 consecutive days in a maze to find an underwater-hidden platform placed in a quadrant, 3 trials per day. On each trial, mice began from one of the quadrants, and ended if the animal climbed on the platform at least for 3 s. If failed to find out the platform within 1 min, mice were leaded to the platform and stayed for 15 s. The spatial memory was examined at day 7. The time to platform (latency), swimming path, duration in target zone and swimming speed were recorded by a video camera (Techman, Chengdu, China), 2 meters from the water surface.

Contextual FCT: Before experiment, mice were allowed to adapt to the environment for 3 min, then the mice were trained by subjecting to foot-shocks last 3 min (0.5 mA, 2 s duration, and 1 min interval). 24 h after, the long-term memory was tested by subjecting back to the conditioning chamber for 3 min, and the freezing time was recorded by a video camera (Techman, Chengdu, China). Complete lack of moving for a minimum of 0.75 second was defined as freezing, and the percent of freezing (%) in each period of time were recorded.

**1.5 Proximity Ligation Assay**

The procedure of experiment was performed according to the manufacturer’s protocol (Sigma). Human embryonic kidney 293T cells grown on glass coverslips were transfected with P301S and KEAP1 vector for 48 h and fixed. The cells were then incubated with antibodies to P301S (Tau5) together with antibody to KEAP1 overnight at 4° C with gentle agitation. PLUS and MINUS secondary PLA probes against rabbit and mouse IgG were added, and the cells were incubated at 37° C for 1 h with gentle agitation, followed by incubation with ligation mix for 30 min at 37° C.

Amplification mix was then applied for 100 min at 37° C. The coverslips were mounted on microscope slides with Doulink Mounting Medium with Dapi, and the cells photographed under a fluorescence microscope (LSM780, Zeiss).

**Figure S1**


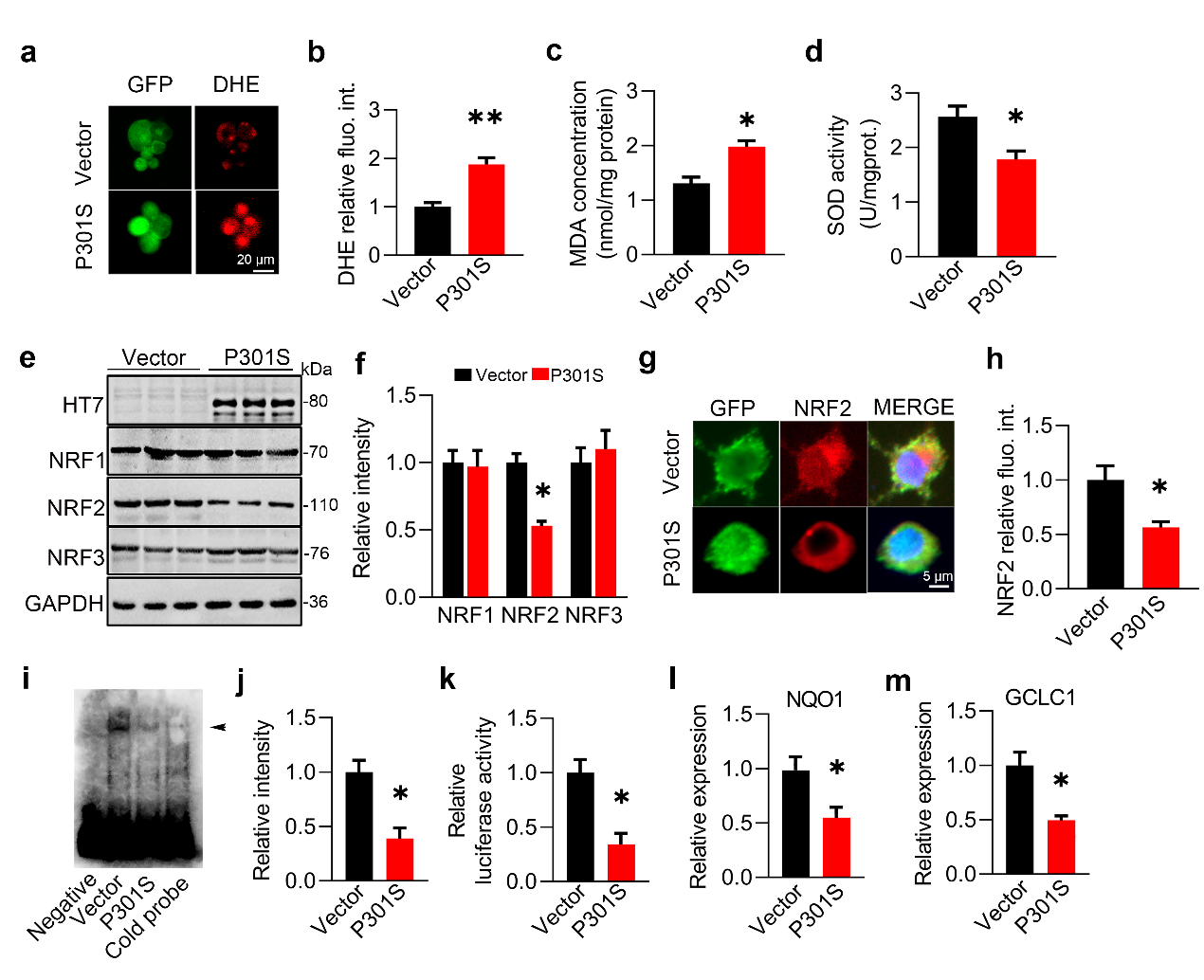


**Supplementary Figure S1. Overexpressing P301S induces oxidative stress and decreases NRF2 without affecting NRF1 and NRF3.** (**a, b**) Overexpressing P301S increased ROS in N2a cells. Unpaired *t*-test, *t* = 4.903 df = 4, *p* = 0.0235, n = 3. (**c, d**) Overexpressing P301S increased MDA concentration (c)and decreased SOD activity (d) in N2a cells. Unpaired *t*-test, [MDA] *t* = 4.307 df = 4, *p* = 0.0126; [SOD] *t* = 3.045 df = 4, *p* = 0.0382, n = 3. (**e, f**) Overexpressing P301S affect protein level of NRF2, but not NRF1 and NRF3, detected by Western blotting. Unpaired *t*-test, [NRF1] *t* = 3.4245 df = 4, *p* = 0.8512; [NRF2] *t* = 4.352 df = 4, *p* = 0.0231; [NRF3] *t* = 4.345 df = 4, *p* = 0.6043, n = 3. (**g, h**) Overexpressing P301S decreased NRF2 protein level in N2a cells detected by immunofluorescence. Unpaired *t*-test, [NRF2] *t* = 3.095 df = 4, *p* = 0.0114, n = 3. (**i,j**) Overexpressing P301S decreased NRF2-DNA binding activity in N2a cells measured by electrophoresis mobility assay (EMSA). Arrow indicates NRF2-DNA complex. Unpaired *t*-test, *t* = 4.168 df = 4, *p* = 0.0140, n = 3. (k) Overexpressing P301S decreased NRF2 transcriptional activity in HEK293 cells detected by dual luciferase reporter assay. Unpaired *t*-test, *t* = 4.178 df = 4, *p* = 0.0139, n = 3. (**l-n**) Overexpressing P301S decreased mRNA level of NQO (l) and GCLC1 (m) in N2a cells. Unpaired *t*-test, [NQO] *t* = 4.642 df = 4, *p* = 0.0421; [GCLC1] *t* = 4.381 df = 4, *p* = 0.0355, n = 3. * *p* < 0.05, ** *p* < 0.01. Data were presented as mean ± SEM.

**Figure S2**


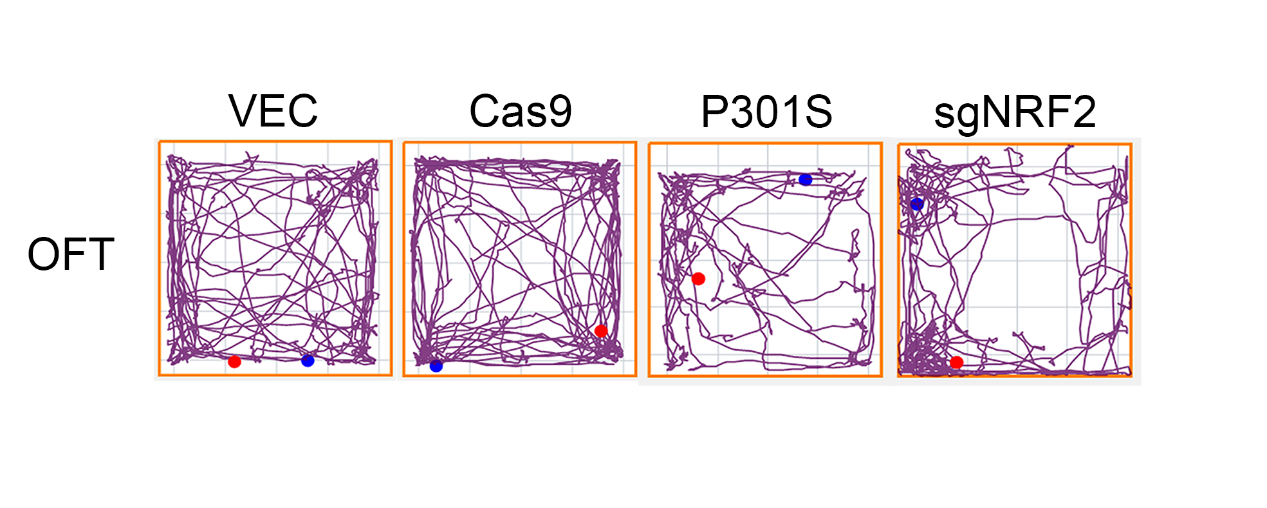


**Supplementary Figure S2. Representative track map shows the time-location of mice traveled in test chambers of open field test (OFT).**

**Figure S3**


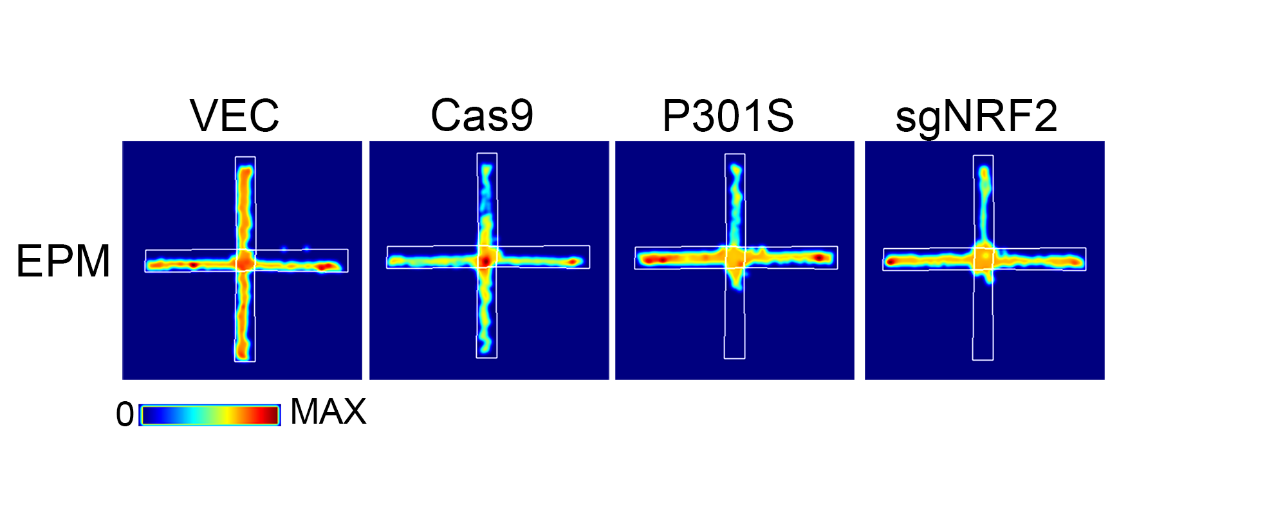


**Supplementary Figure S3. Representative heat map shows the time-location of mice traveled in test chambers of elevated plus maze (EPM).**

**Figure S4**

**
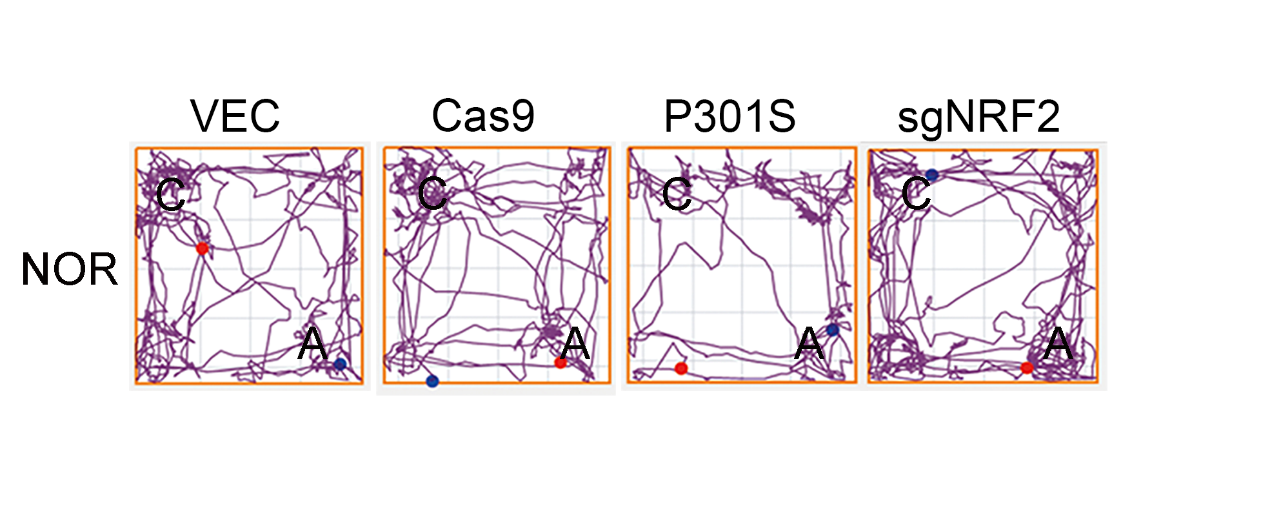
**

**Supplementary Figure S4. Representative track map shows the time-location of mice traveled in test chambers of novel object recognition (NOR).**

**Figure S5**

**
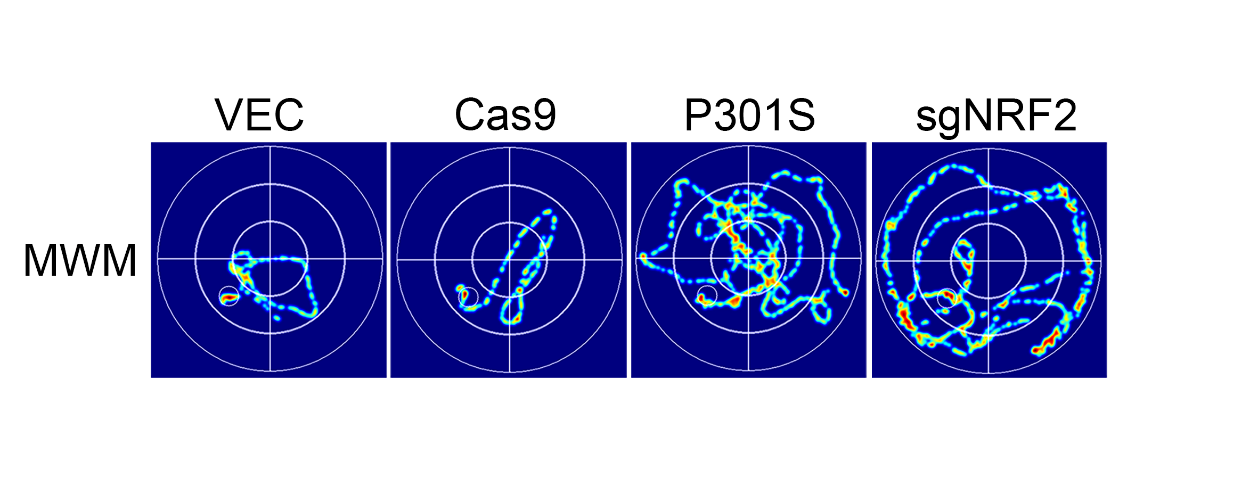
**

**Supplementary Figure S5. Representative track map shows the time-location of mice traveled in test chambers of Morris water maze (MWM).**

**Figure S6**


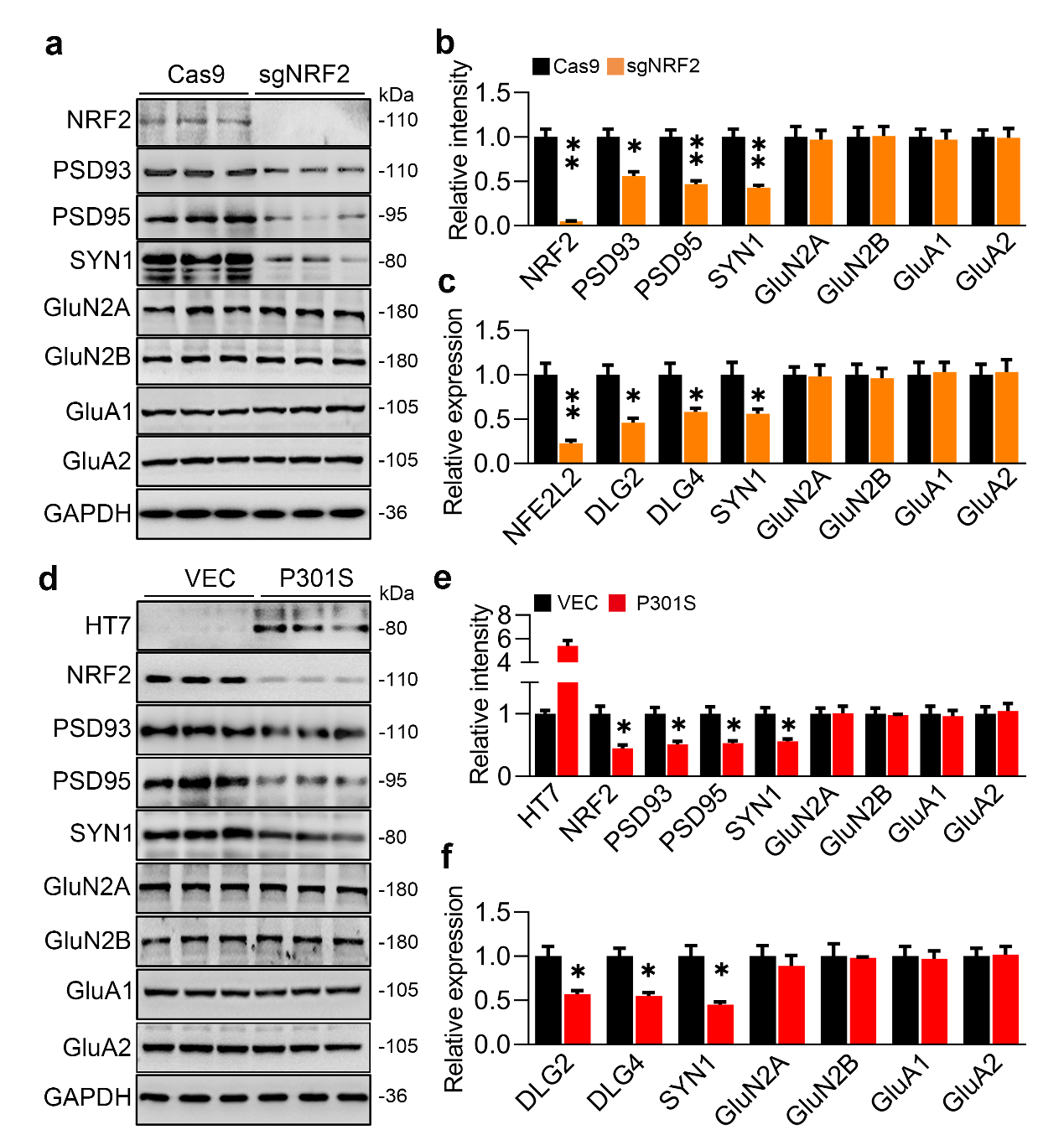


**Supplementary Figure S6. Knockdown NRF2 decreases multiple synaptic proteins**. (**a, b**) Knockdown NRF2 in N2a cells by transfecting with plasmid coding sgNRF2 decreased protein levels of synaptic proteins detected by Western blotting. Unpaired *t*-test, [NRF2] *t* = 10.55 df = 4, *p* = 0.0005; [PSD93] *t* = 4.334 df = 4, *p* = 0.0123; [PSD95] *t* = 5.451 df = 4, *p* = 0.0055; [SYN1] *t* = 3.096 df = 4, *p* = 0.0041, n = 3. (**c**) Knockdown NRF2 in N2a cells by transfecting with plasmid coding sgNRF2 decreased mRNA levels of synaptic proteins detected by qPCR. Unpaired *t*-test, [NFE2L2] *t* = 5.771 df = 4, *p* = 0.0045; [DLG2] *t* = 4.469 df = 4, *p* = 0.0111; [DLG4] *t* = 3.088 df = 4, *p* = 0.0366; [SYN1] *t* = 2.960 df = 4, *p* = 0.0416, n = 3. (**d, e**) Overexpressing P301S in N2a cells by transfecting with plasmid decreased protein levels of synaptic proteins detected by Western blotting. Unpaired *t*-test, [NRF2] *t* = 4.231 df = 4, *p* = 0.0134; [PSD93] *t* = 4.490 df = 4, *p* = 0.0101; [PSD95] *t* = 4.047 df = 4, *p* = 0.0155; [SYN1] *t* = 4.324 df = 4, *p* = 0.0124, n = 3. (**f**) Overexpressing P301S in N2a cells by transfecting with plasmid decreased mRNA levels of synaptic proteins examined by qPCR. Unpaired *t*-test, [DLG2] *t* = 3.662 df = 4, *p* = 0.0215; [DLG4] *t* = 4.412 df = 4, *p* = 0.0116; [SYN1] *t* = 4.438 df = 4, *p* = 0.0114, n = 3. * *p* < 0.05, ** *p* < 0.01. Data were presented as mean ± SEM.

**Figure S7**

**
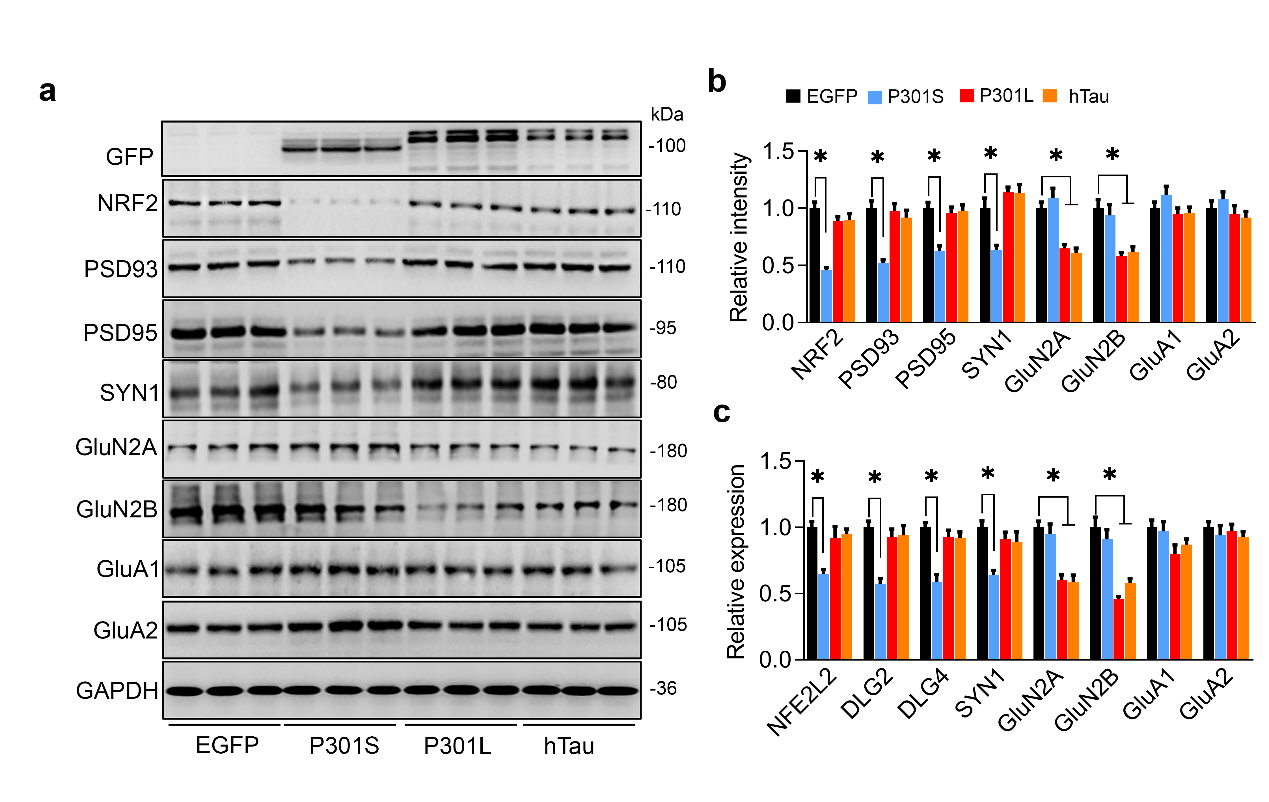
**

**Supplementary Figure S7.** **The effect of overexpressing wild-type or mutated hTau on NRF2 and multiple synaptic proteins**. (**a-c**) Overexpressing wild-type or mutated hTau in N2a cells by transfecting with plasmid decreased protein and mRNA levels of NRF2 and synaptic proteins detected. One-way ANOVA, for pane b [NRF2] F(3, 8) = 6.764, *p* = 0.0145; [PSD93] F(3, 8) = 9.546, *p* = 0.0433; [PSD95] F(3, 8) =8.657, *p* = 0.0346; [SYN1] F(3, 8) =6.095, *p* = 0.0356; [GluN2A] F(3, 8) = 13.23, *p* = 0.0232; [GluN2B] F(3, 8) = 11.32, *p* = 0.0271, n = 3. for pane c [NFE2L3] F(3, 8) = 11.21, *p* = 0.0235; [DLG2] F(3, 8) = 11.34, *p* = 0.0253; [DLG4] F(3, 8) = 12.47, *p* = 0.0246; [SYN1] F(3, 8) = 16.095, *p* = 0.0167; [GluN2A] F(3, 8) = 9.453, *p* = 0.0220; [GluN2B] F(3, 8) = 7.875, *p* = 0.0271, n = 3. * *p* < 0.05, ** *p* < 0.01. Data were presented as mean ± SEM.

**Figure S8**


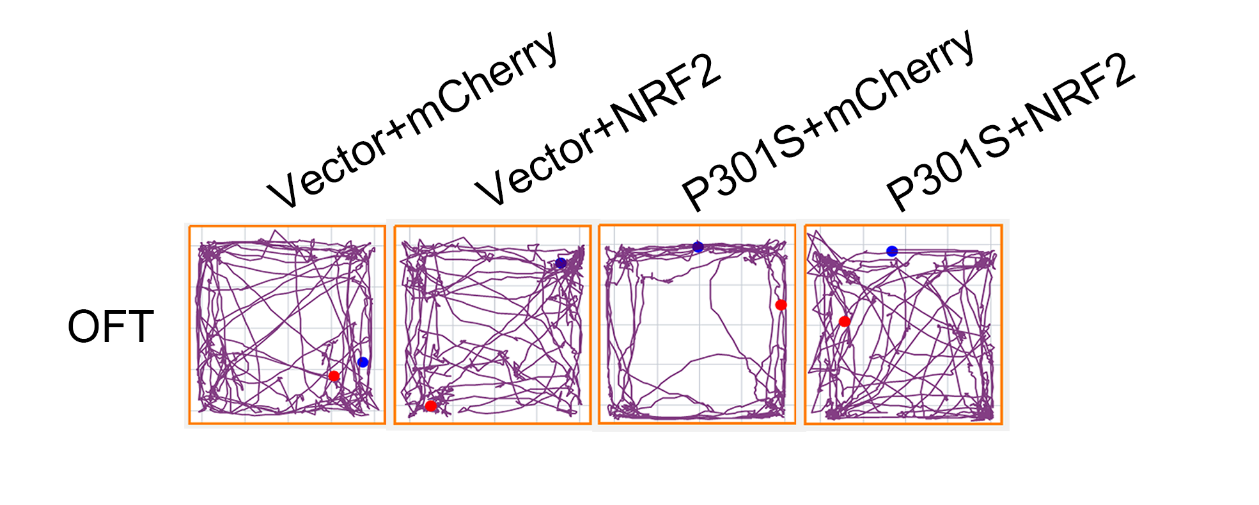


**Supplementary Figure S8. Representative track map shows the time-location of mice traveled in test chambers of OFT.**

**Figure S9**


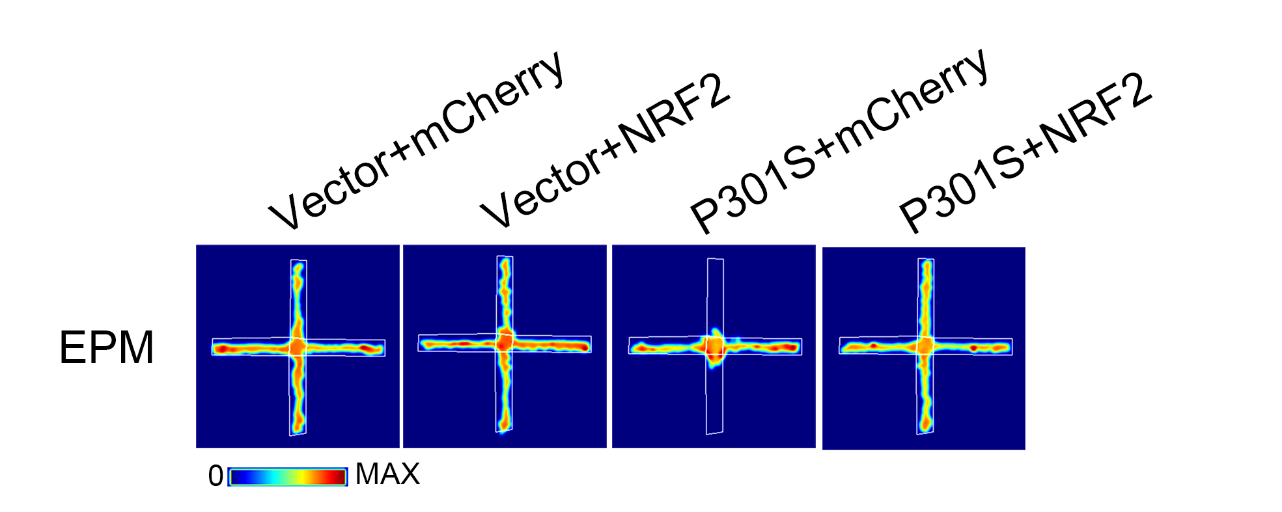


**Supplementary Figure S9. Representative heat map shows the time-location of mice traveled in test chambers of EPM.**

**Figure S10**


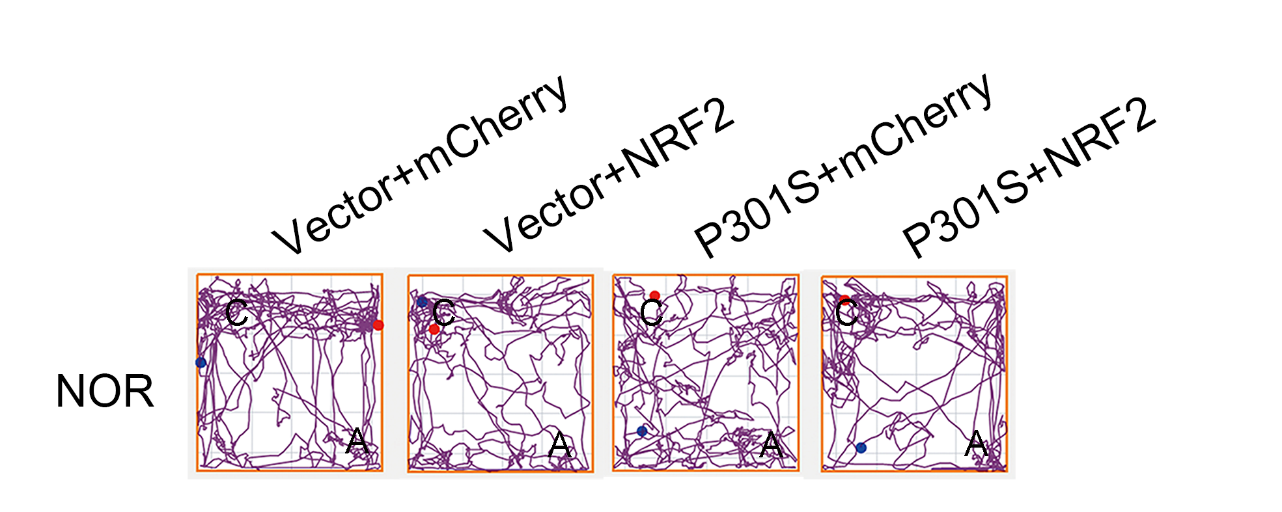


**Supplementary Figure S10. Representative track map shows the time-location of mice traveled in test chambers of NOR.**

**Figure S11**


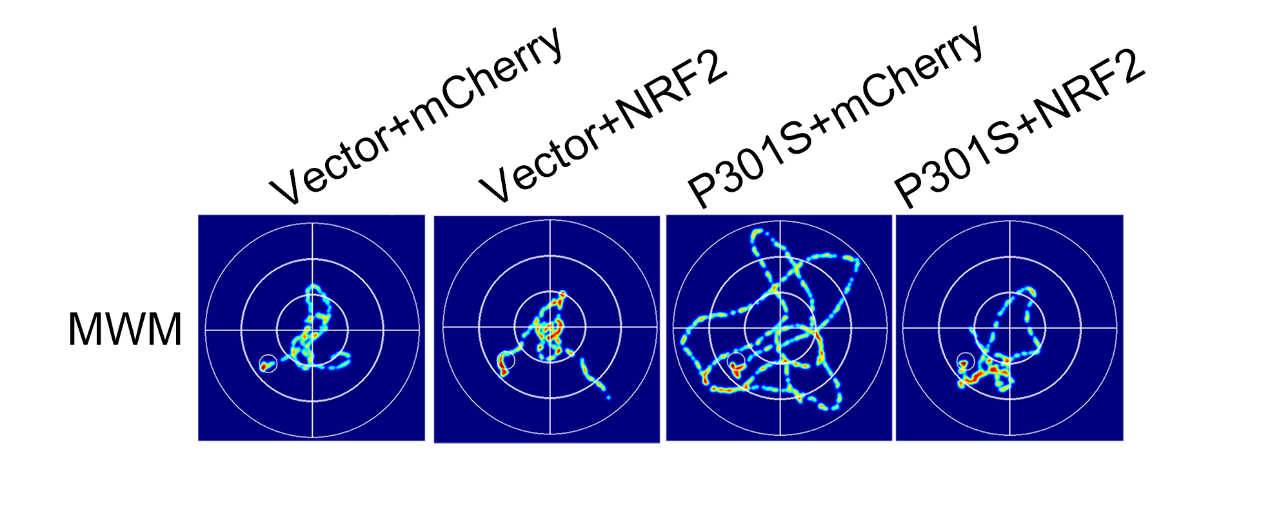


**Supplementary Figure S11. Representative track map shows the time-location of mice traveled in test chambers of MWM.**

**Figure S12**

**
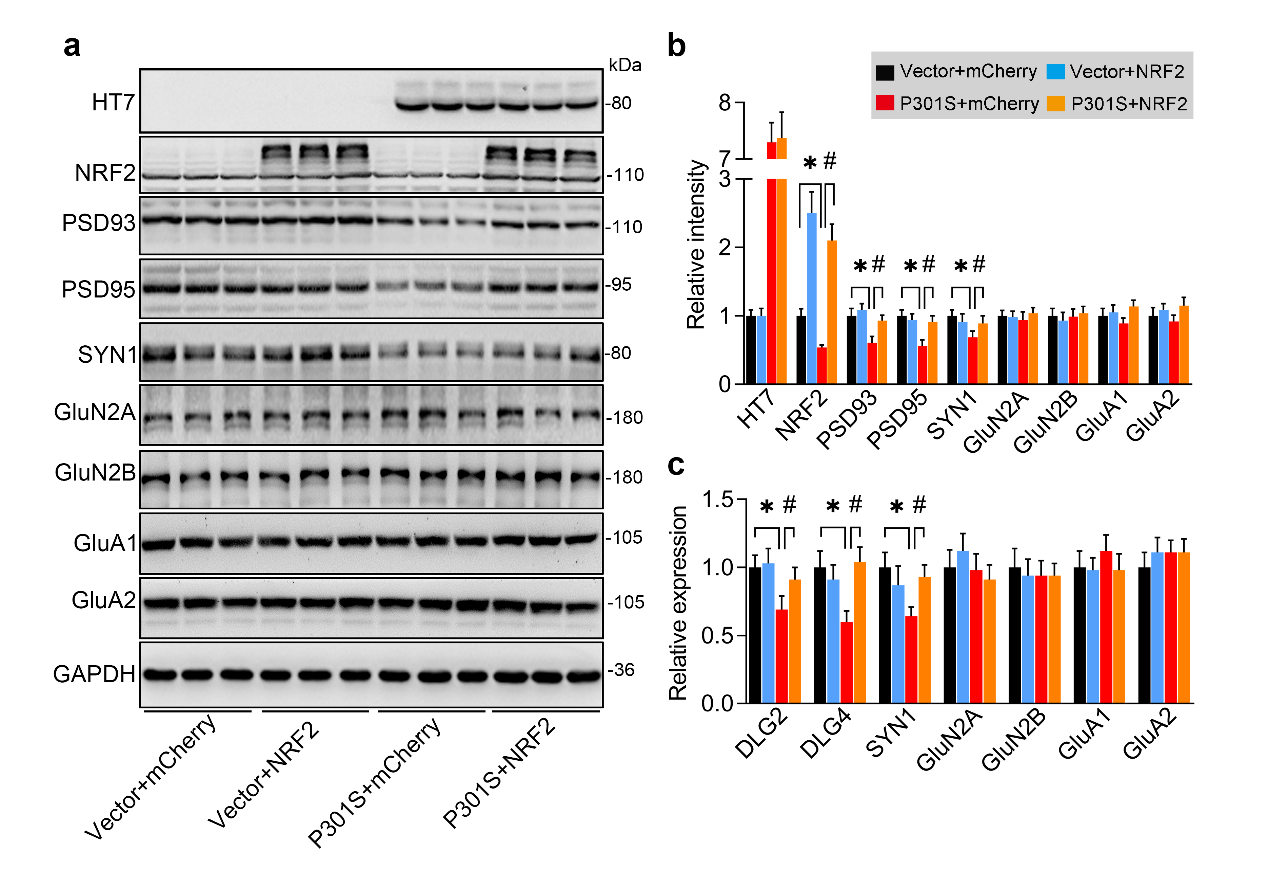
Supplementary Figure S12. Overexpressing NRF2 ameliorates P301S-induced synaptic proteins reduction.** (**a, b**) Overexpressing NRF2 reversed protein levels of synaptic proteins in N2a cells transfected with P301S plasmids detected by Western blotting. One-way ANOVA, [NFR2] F(3, 8) = 69.91, *p* < 0.0001; [PSD93] F(3, 8) = 7.263, *p* = 0.0113; [PSD95] F(3, 8) = 5.528, *p* = 0.0237; [SYN1] F(3, 8) = 5.093, *p* = 0.0292, n = 3. (**c**) Overexpressing NRF2 reversed mRNA levels of synaptic proteins in N2a cells transfected with P301S plasmids detected by qPCR. One-way ANOVA, [DLG2] F(3, 8) = 11.13, *p* = 0.0032; [DLG4] F(3, 8) = 5.075, *p* = 0.0295; [SYN1] F(3, 8) = 4.649, *p* = 0.0365, n = 3. * *p* < 0.05 *vs* VEC +mCherry; # *p* < 0.05, ## *p* < 0.01 vs P301S + mCherry. Data were presented as mean ± SEM.

**Figure S13**


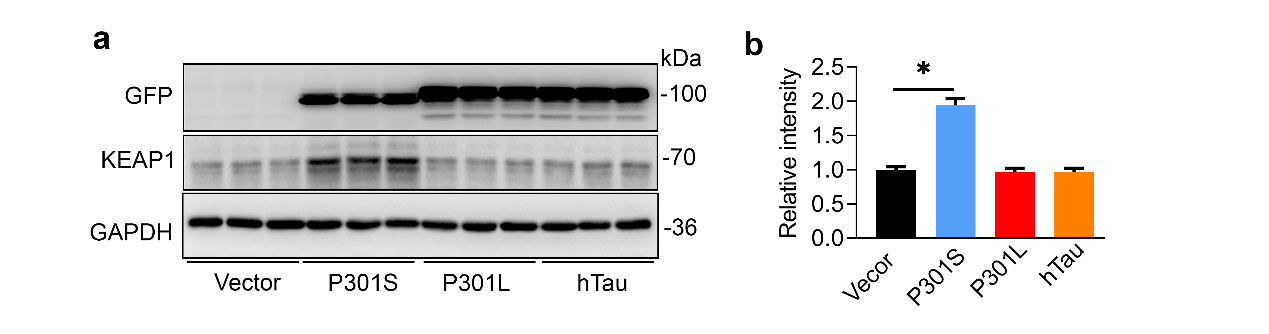


**Supplementary Figure S13. The vulnerability of KEAP1 to P301S.** (**a, b**) Overexpressing P301S, but not wild-type or P301L hTau, enhanced KEAP1’ protein level. One-way ANOVA, F(3, 8) = 32.12, *p =* 0.0032. * *p* < 0.05. Data were presented as mean ± SEM.

**Figure S14**


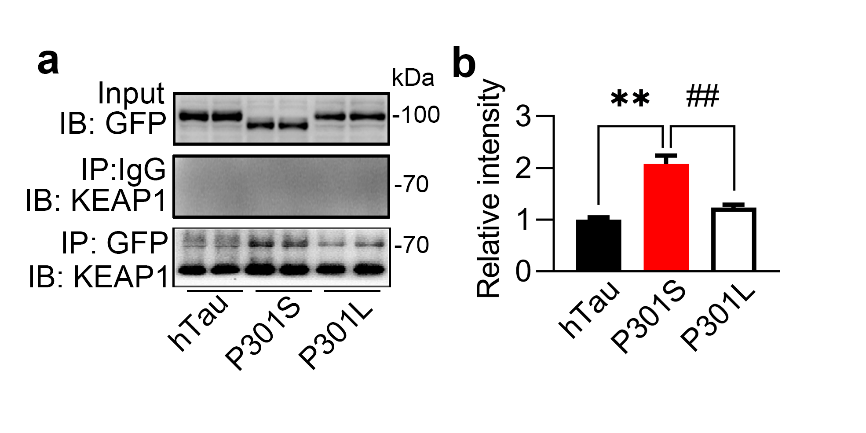


**Supplementary Figure** **S14.** **P301S shows the strongest interaction with KEAP1 compared with wild-type hTau and P301L hTau.** (**a, b**) Overexpressing wild-type hTau, P301S or P301L plasmids (GFP-label) in N2a cell for 48 h, cells were harvested and GFP antibody was added for immunoprecipitation (IP), then analyzed by Western blotting using GFP and KEAP1 antibody. One-way ANOVA, F(2, 6) = 32.32, *p* = 0.0006, n = 3.** *p* < 0.01 *vs* hTau; ## *p* < 0.01 *vs* P301L. Data were presented as mean ± SEM. IB: immunoblotting, IP: immunoprecipitation.

**Figure S15**

**
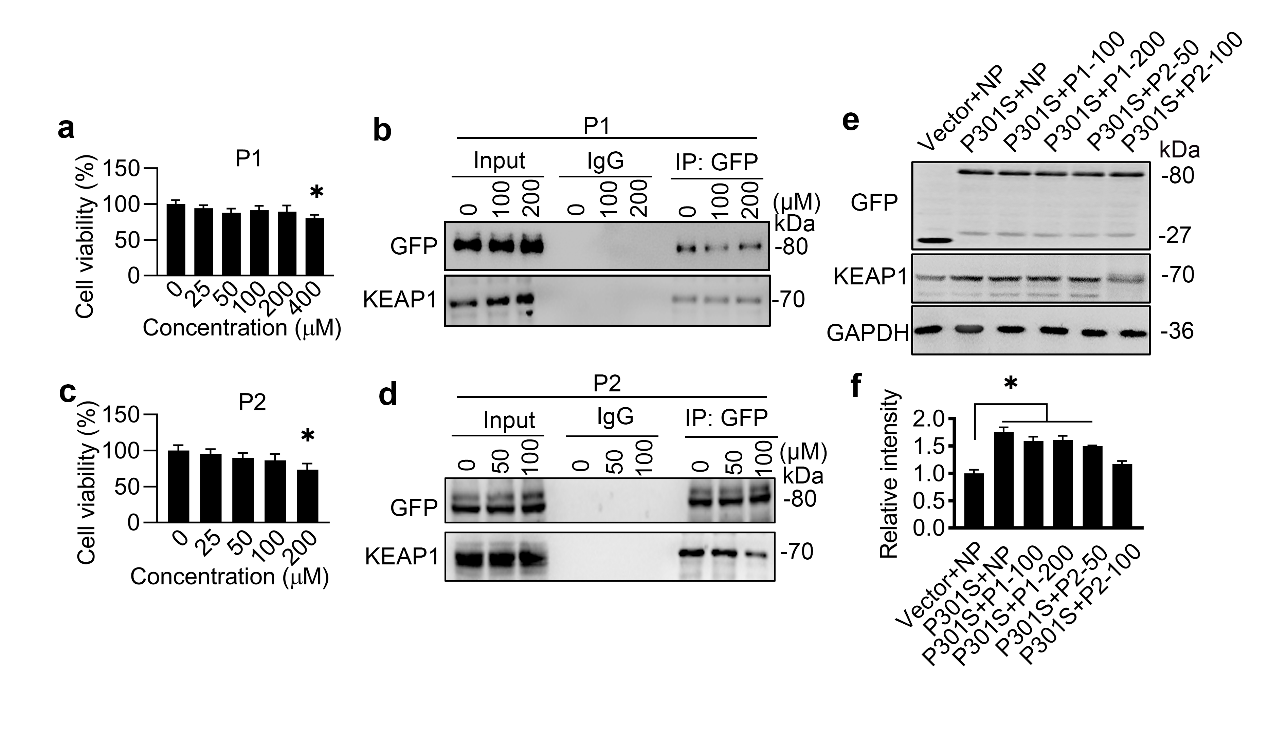
Supplementary Figure S15. The custom-designed peptide significantly decreases P301S-KEAP1 interaction and ameliorates P301S-induced anxiety.** (**a**) CCK8 assayed cytotoxicity of custom-designed peptide P1. One-way ANOVA, F(5, 12) = 1.177, *p* = 0.04, n = 3. * *p* < 0.05 *vs* 0. (**b**) Custom-design peptide P1 didn’t blocks interaction between P301S and KEAP1 as measured by IP and Western blotting. (**c**) CCK8 assayed cytotoxicity of custom-designed peptide P2. One-way ANOVA, F(4, 10) = 1.710, *p* = 0.3757, n = 3. * *p* < 0.05 *vs* 0. (**d**) Custom-design peptide P2 blocks interaction between P301S and KEAP1 as measured by IP and Western blotting. (**e, f**) P2 decreased protein level of KEAP1 in N2a cells transfected with P301S plasmids detected by Western blot. One-way ANOVA, F(5, 12) = 5.299. *p* = 0.0065. n = 3. * *p* < 0.05Data were presented as mean ± SEM.

**Figure S16**


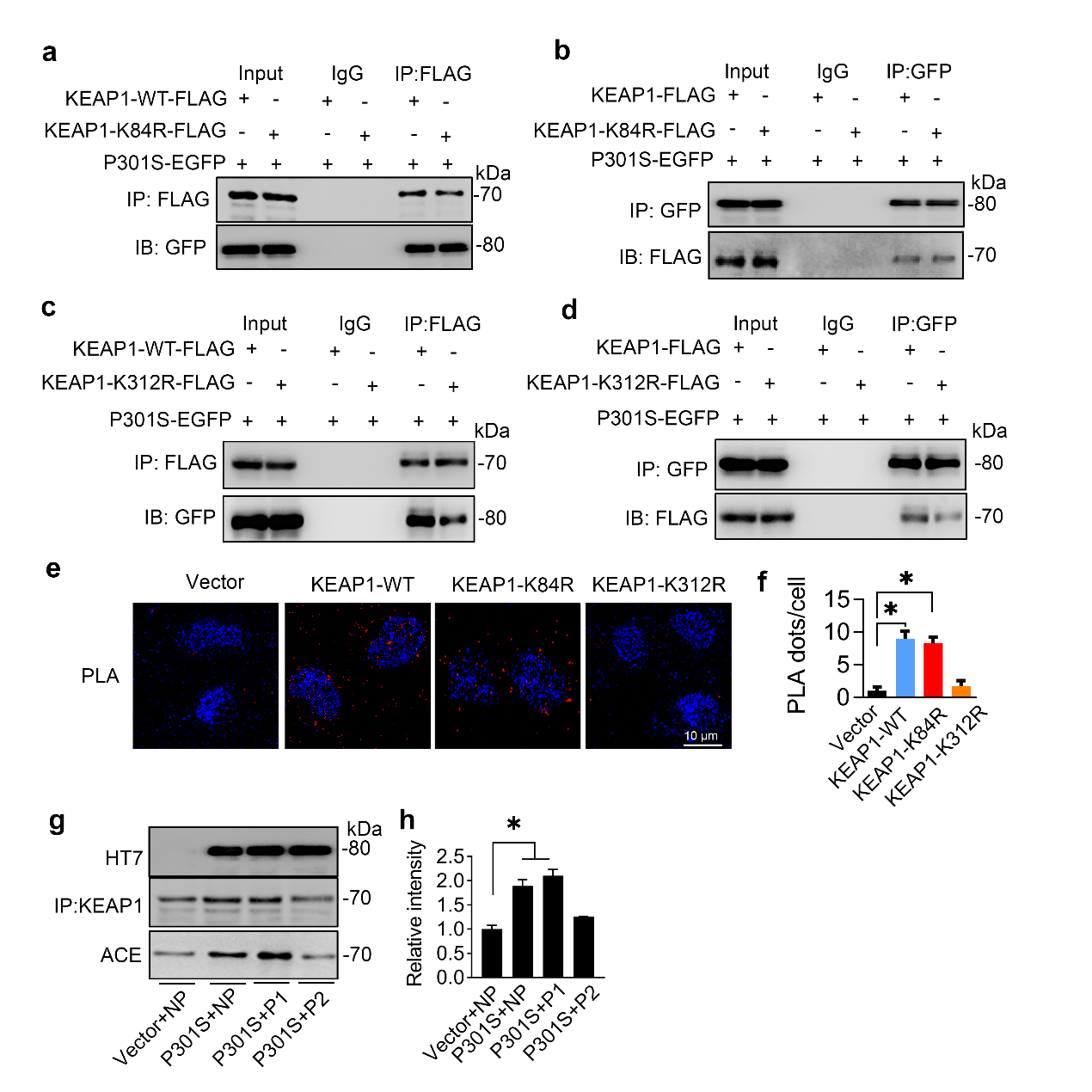


**Supplementary Figure S16. P301S binds to K312 of KEAP1.** (**a, b**) K84R mutation didn’t attenuates association between P301S and KEAP1 as measured by IP and WB. (**c, d**) K84R mutation didn’t attenuates association between P301S and KEAP1 as measured by IP and WB. (**e, f**) K312R mutation decreases association between P301S and KEAP1 as Proximity ligation assay (PLA). One-way ANOVA, One-way ANOVA, F(3, 8) = 7.765, *p* = 0.0043, n = 3. (**g, h**) P2 decreased ACE level of KEAP1 in N2a cells transfected with P301S plasmids detected by Western blotting. One-way ANOVA, F(3, 8) = 9.354, *p* = 0.0026. n = 3. * *p* < 0.05. Data were presented as mean ± SEM.

**Figure S17**


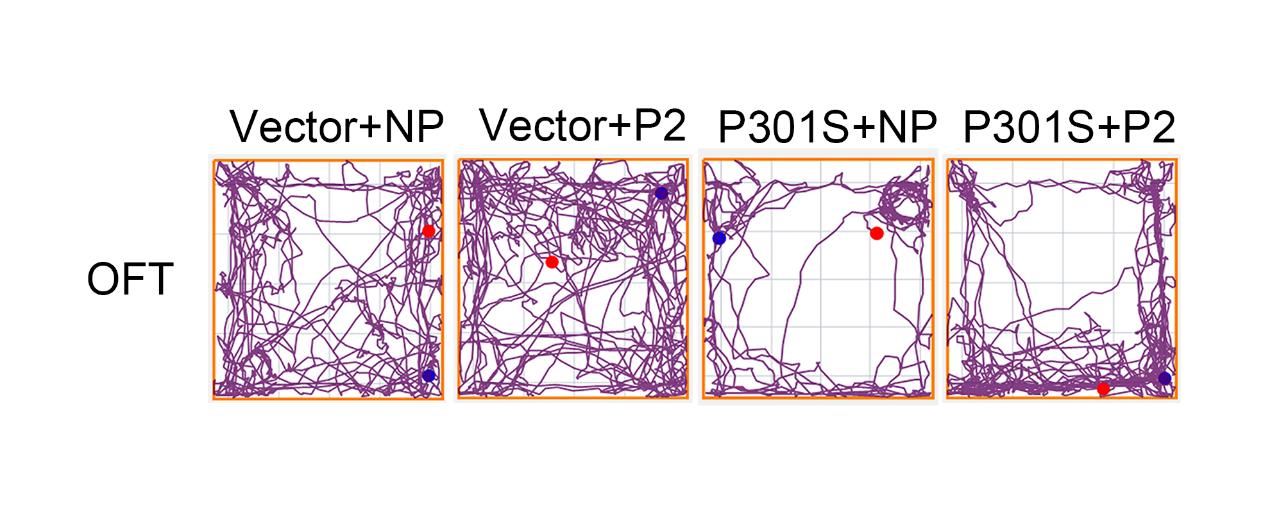


**Supplementary Figure 17. Representative track map shows the time-location of mice traveled in test chambers of OFT.**

**Figure S18**


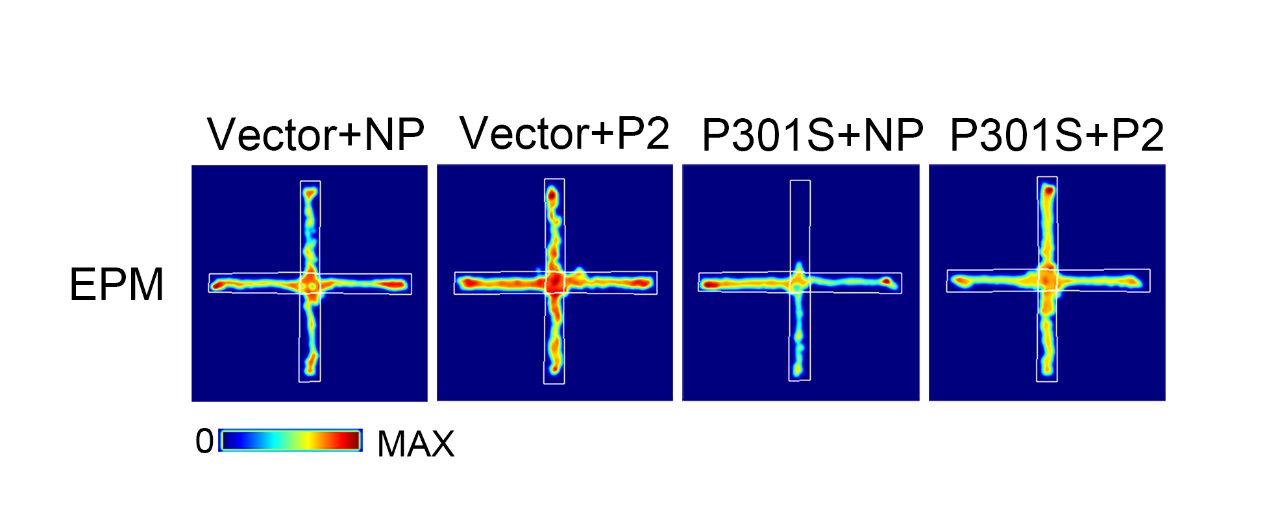


**Supplementary Figure 18. Representative heat map shows the time-location of mice traveled in test chambers of EPM.**

**Figure S19**


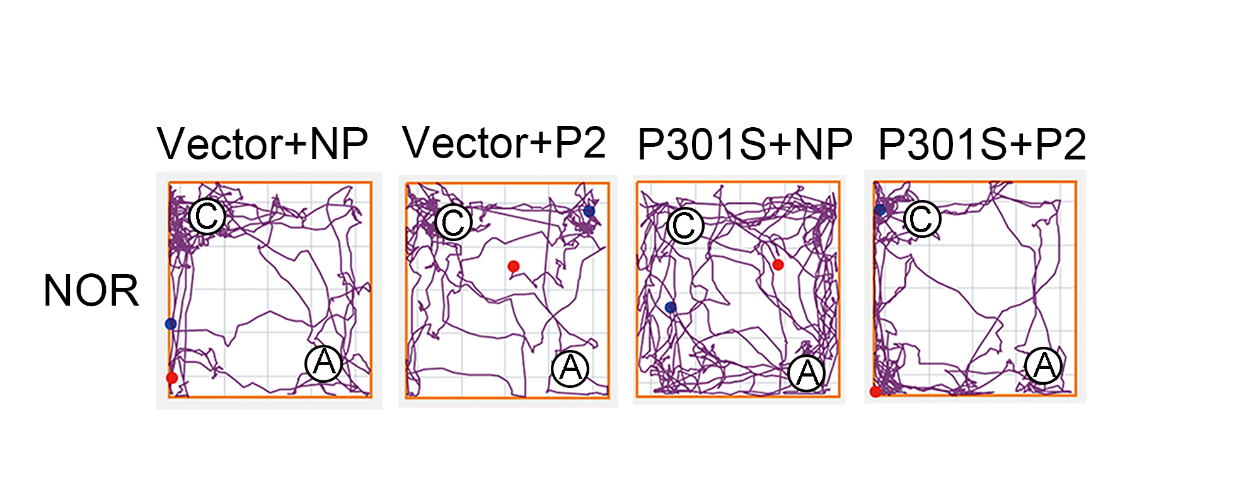


**Supplementary Figure 19. Representative heat map shows the time-location of mice traveled in test chambers of NOR.**

**Figure S20**


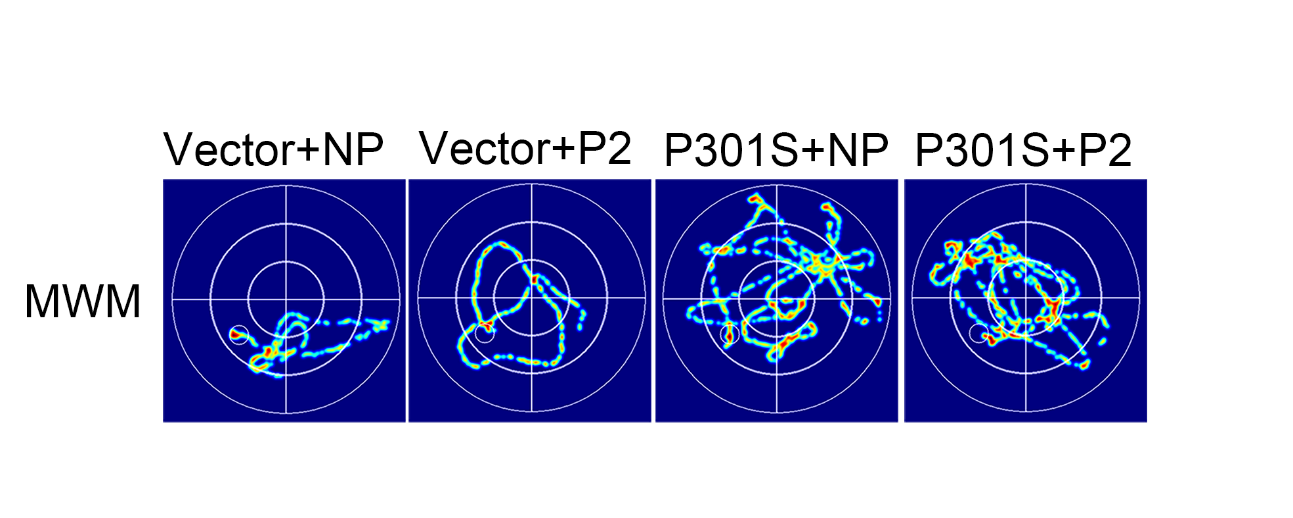


**Supplementary Figure 20. Representative track map shows the time-location of mice traveled in test chambers of MWM.**

**Figure S21**


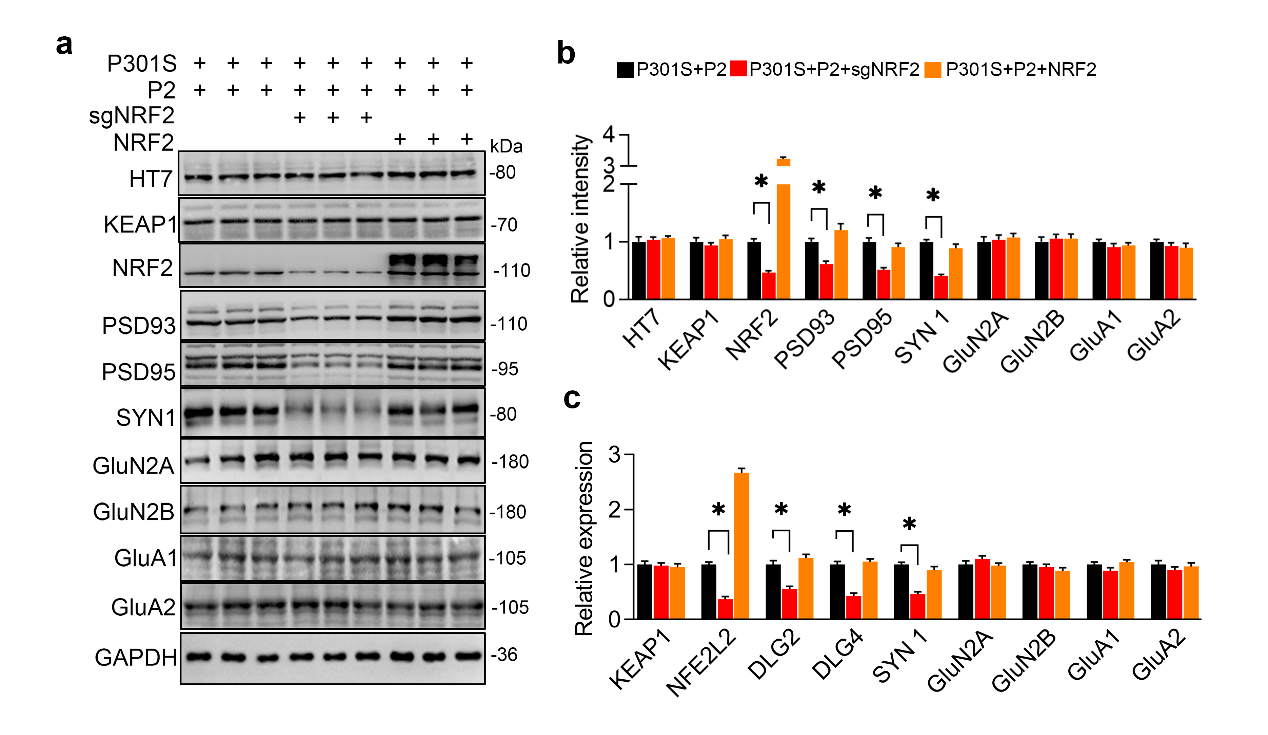


Supplementary Figure S21. Knockdown NRF2 blocked P2’s protective effect against P301S-hTau. (**a, b**) Knockdown NRF2 blocked P2’s protective effect against P301S-hTau impairment on proteins level of synapse. One-way ANOVA, [NRF2] F(2, 6) = 788.2, *p* < 0.002; [PSD93] F(2, 6) = 14.56, *p* = 0.0351; [PSD95] F(2, 6) = 18.26, *p* = 0.0028; [SYN1] F(2, 6) = 38.43, *p* = 0.0004, n = 3. (**c**) Knockdown NRF2 blocked P2’s protective effect against P301S-hTau impairment on mRNA level of synapse. One-way ANOVA, [NFE2L2] F(2, 6) = 392.7, *p* < 0.0001; [DLG2] F(2, 6) = 22.35, *p* = 0.0170; [DLG4] F(2, 6) = 38.71, *p* = 0.004; [SYN1] F(2, 6) = 29.89, *p* = 0.008, n = 3. * *p* < 0.05. Data were presented as mean ± SEM.

| **Antibody** | **Host** | **Manufacturer** | **Cat #** | **Dilution** | | | | |
| --- | --- | --- | --- | --- | --- | --- | --- | --- |
|  |  |  |  | **WB** | **ICC** | **IHC** | **IP** | **ChIP** |
| HT7 antibody | mouse | Thermofisher | NM1000 | 1:1000 |  |  |  |  |
| KEAP1 antibody | rabbit | Proteintech | 10503-2-AP | 1:1000 | 1:200 | 1:100 | 1:50 |  |
| NRF2 antibody | rabbit | Proteintech | 16396-1-AP | 1:1000 | 1:100 |  |  | 1:20 |
| LaminB1 antibody | rabbit | Proteintech | 12987-1-AP | 1:1000 |  |  |  |  |
| GFP antibody | rabbit | Proteintech | 50403-2-AP | 1:2000 |  |  | 1:50 |  |
| Flag antibody | mouse | Proteintech | 66008-3-Ig | 1: 1000 | 1:200 |  | 1:50 |  |
| GAPDH antibody | mouse | Proteintech | 60004-1-Ig | 1:10000 |  |  |  |  |
| β actin antibody | mouse | Proteintech | 60008-1-Ig | 1:10000 |  |  |  |  |
| PSD93 antibody | rabbit | Abclonal | A19669 | 1:1000 |  |  |  |  |
| PSD95 antibody | rabbit | Abclonal | A0131 | 1:1000 |  |  |  |  |
| SYN1 antibody | rabbit | Abclonal | A17362 | 1:1000 |  |  |  |  |
| GluN2A antibody | rabbit | Abclonal | A19089 | 1:1000 |  |  |  |  |
| GluN2B antibody | rabbit | Abclonal | A3056 | 1:1000 |  |  |  |  |
| GluA1 antibody | rabbit | Abclonal | A11643 | 1:1000 |  |  |  |  |
| GluA2 antibody | rabbit | Abclonal | A11316 | 1:1000 |  |  |  |  |
| Ubiquitin antibody | mouse | Cell signaling | 3936 | 1:1000 |  |  |  |  |
| Acetylated-lysine antibody | mouse | Cell signaling | 9441 | 1:1000 |  |  |  |  |
| Tau5 antibody | mouse | Abcam | ab80579 |  | 1:100 |  |  |  |
| CoraLite-488 Goat-anti-mouse IgG(H+L) IgG | Goat | Proteintech | SA00013-1 | 1:200 |  |  |  |  |
| CoraLite-488 Goat-anti-rabbit IgG(H+L) IgG | Goat | Proteintech | SA00013-2 | 1:200 |  |  |  |  |
| CoraLite-594 Goat-anti-mouse IgG(H+L) IgG | Goat | Proteintech | SA00013-3 | 1:200 |  |  |  |  |
| CoraLite-594 Goat-anti-rabbit IgG(H+L) IgG | Goat | Proteintech | SA00013-4 | 1:200 |  |  |  |  |
| Peroxidase-Conjugated Goat-anti-Mouse IgG(H+L) IgG | Goat | Beyotime | A0216 | 1:2000 |  |  |  |  |
| Peroxidase-Conjugated Goat-anti-Rabbit IgG(H+L) IgG | Goat | Beyotime | A0208 | 1:2000 |  |  |  |  |

**Table S1** Antibody list

**Table S2** List of qPCR Primers

| Name | Sequence |
| --- | --- |
| DLG2 | F: TTGTGCACTCCGGACTAACG |
| DLG2 | R: GCACAAGTTCTGGCCCTCTT |
| DLG4 | F: AGTGCTGCACAGTGAT CTCTTC |
| DLG3 | R: CGTGTCTTCATCTTGGTAGCG |
| SYN1 | F: AGCTCAACAAATCCCAGTCTCT |
| SYN1 | R: CGGATGGTCTCAGC TTTCAC |
| GluN2A | F: ACGTGACAGAACGCGAACTT |
| GluN2A | R: TCAGTGCGGTTCATCAATAACG |
| GluN2B | F: GCCATGAA CGAGACTGACCC |
| GluN2B | R: GCTTCCTGGTCCGTGTCATC |
| GluA1 | F: TCCCCAACAATATCCAGATAG |
| GluA | R: AAGCCGCATGTTCCTGTGATT |
| GluA2 | F: TTCTCCTGTTTTATGGGGACTGA |
| GluA2 | R: CTACCCGAAATGCACTGTATTCT |
| NFE2L2 | F: CAGTCAGCGACGGAAAGAGT |
| NFE2L2 | R: ACCTGGGAGTAGTTGGCAGA |
| KEAP1 | F: GATGGGCAGGACCAGTTGAA |
| KEAP1 | R: CCGAGGACGTAGATCTTGCC |
| ACTB | F: GGCTGTATTCCCCTCCATCG |
| ACTB 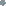 | R: CCAGTTGGTAACAATGCCATGT |

**Table S3** list of ChIP primers

| Name | | Sequence |
| --- | --- | --- |
| ARE1 | DLG2 | F: GACGACACTTTCTGAACGCTTA |
| ARE1 | DLG2 | R: AGCAGTGCTGATGCTCTCTG |
| ARE2 | DLG2 | F: GATGCAATCCCCTGGTAAAG |
| ARE2 | DLG2 | R: GAGGATTCAGCATCCGAAAG |
| ARE3 | DLG2 | F: GCTCTCACTCAGTGCCTTCA |
| ARE3 | DLG2 | R: GATCAGCTTCTCAGCACAGC |
| ARE1 | SYN1 | F: ACTTTTGAACCCCCAAAACC |
| ARE1 | SYN1 | R: CCTCTTTGCAGACAGGAAGC |
| ARE2 | SYN1 | F: CTCTTTTCTTTGCCCGACAG |
| ARE2 | SYN1 | R: TTGCCCTTCCCAGATACTTG |
| ARE3 | SYN1 | F: ACGAGAGGTGCAAGATAGGG |
| ARE3 | SYN1 | R: AAGGGGCAGTGGGTCTGT |
| ARE1 | DLG4 | F: TCCTCTGCCTAAGAGCAACG |
| ARE1 | DLG4 | R: TGTCATCAGGGACCCACATA |
